# Transcript and Protein Profiling Provides Insights Into the Molecular Mechanisms of Harvesting-Induced Latex Production in Rubber Tree

**DOI:** 10.3389/fgene.2022.756270

**Published:** 2022-02-10

**Authors:** Yujie Fan, Jiyan Qi, Xiaohu Xiao, Heping Li, Jixian Lan, Yacheng Huang, Jianghua Yang, Yi Zhang, Shengmin Zhang, Jun Tao, Chaorong Tang

**Affiliations:** ^1^ Natural Rubber Cooperative Innovation Center of Hainan Province and Ministry of Education of PRC, Hainan University, Haikou, China; ^2^ Rubber Research Institute, Chinese Academy of Tropical Agricultural Sciences, Haikou, China

**Keywords:** *Hevea brasiliensis*, cDNA-AFLP, 2-DE, gene and protein expression, harvesting, latex production

## Abstract

Natural rubber, an important industrial raw material with wide applications, is harvested in the form of latex (cytoplasm of rubber-producing laticifers) from *Hevea brasiliensis* (para rubber tree) by the way of tapping. Conspicuous stimulation on latex production is observed for the first few tappings conducted on virgin (untapped before) or resting (tapped before but no tapping for a period) rubber trees. To understand the underlying mechanisms, an integrative analysis of the latex transcriptome and proteome was conducted on virgin or resting *Hevea* trees for the first five tappings. A total of 505 non-redundant differentially expressed (DE) transcript-derived fragments (TDFs) were identified by silver-staining cDNA-AFLP, with 217 exhibiting patterns of upregulated, 180 downregulated and 108 irregularly-regulated. Meanwhile, 117 two dimensional gel electrophoresis DE-protein spots were isolated and subjected to mass spectrometry analysis, with 89 and 57 being successfully identified by MALDI-TOF and MALDI-TOF/TOF, respectively. About 72.5% DE-TDFs and 76.1% DE-proteins were functionally annotated and categorized. Noteworthily, most of the DE-TDFs implicated in sugar transport and metabolism as well as rubber biosynthesis were upregulated by the tapping treatment. The importance of sugar metabolism in harvesting-induced latex production was reinforced by the identification of abundant relevant DE-protein spots. About 83.8% of the randomly selected DE-TDFs were validated for expression patterns by semi-quantitative RT-PCR, and an 89.7% consistency for the 29 latex regeneration-related DE-TDFs examined by quantitative RT-PCR analysis. In brief, our results reveal extensive physiological and molecular changes in *Hevea* laticifers incurred by the tapping treatment, and the vast number of DE genes and proteins identified here contribute to unraveling the gene regulatory network of tapping-stimulated latex production.

## Introduction

Natural rubber (*cis*-1, 4-polyisoprene, NR) is an elastomer with superior properties that cannot be completely replaced by petroleum-derived synthetic rubber and is used as an important industrial raw material. Due to the advantages of good quality, high yield, and easiness for harvesting, *Hevea brasiliensis* (para rubber tree, *Hevea* thereafter) has become the sole commercial NR source among the 2, 500 NR-bearing plant species (Van Beilen and Poirier, 2007). *Hevea* trees need warm and humid climate conditions for normal growth and NR production yet vulnerable to typhoon, thus confining its planting to restricted tropical areas (Huang, 2001).

*Hevea* rubber yield is affected mainly by three factors: duration of latex flow after tapping, the capability of latex regeneration between two consecutive tappings, and the ability of laticifer differentiation in bark cambium (d’Auzac et al., 1997; Hao et al., 2004). In the trunk bark of tapped *Hevea* trees, the number of laticifer rings is one to three times more than that observed in un-tapped trees, indicating the stimulating effect of tapping on laticifer differentiation (Hao and Wu, 1982). Meanwhile, both mechanical injury and jasmonic acid induce laticifer differentiation, latex regeneration and production (Hao and Wu, 2000; Sae-Lim et al., 2019). Cytologically, latex is the cytoplasm of the laticifers, 90% of the dry weight as rubber hydrocarbon (NR) (Chrestin et al., 1997). To sustain NR productivity, in regularly tapped *Hevea* trees, the expelled latex must be efficiently regenerated before the next tapping, usually with an interval of 2–3 days (d’Auzac et al., 1997).

After tapping, the latex flowing out of the laticifers includes cis-polyisoprene particles (rubber particles), lysosomal microvacuoles (lutoids), plastid-like Frey-Wyssling complexes, sugars, organic acids, nucleic acids, and proteins/enzymes (d'Auzac et al., 1997). A positive correlation exists between sucrose content and latex yield, and the control of sucrose metabolism has been intensively studied in relation to latex production (Tupý, 1969; Tupý, 1985; Tupý, 1989). Expression of the responsible sucrose transporter, HbSUT3, for sucrose loading into laticifers is induced by the treatments of ethylene and tapping, both bolstering the latex yield (Tang et al., 2010). In the latex, an alkaline/neutral invertase is responsible for cleaving sucrose into hexose sugars that are then exploited in subsequent latex production (Tupý, 1989). Expression and enzymatic activity of the relevant invertase gene, *HbNIN2*, correlate positively with the latex yield (Liu et al., 2015). Rubber particles are a kind of organelle suspended in latex where rubber biosynthesis takes place and subsequently stores, with proteins of rubber elongation factor (REF)/small rubber particle protein (SRPP) as important rubber-synthesizing participants (Cornish, 1993; Oh et al., 1999; Singh et al., 2003; Tang et al., 2016). Priya et al. (2007) observed a positive correlation between REF mRNA abundance and the latex yielding levels of different *Hevea* clones. After Ethrel (2-chloroethylphosphonic acid, an ethylene releaser) bark treatment, a marked increase in transtonoplast ΔpH within *Hevea* laticifers was observed consisting of one of the major mechanisms of ethylene stimulation on latex yield (Amalou et al., 1992). Together, these studies suggest that latex production, with NR biosynthesis as the core activity, involves a complex regulatory network of gene expression, multi-enzyme reaction and physio-biochemical processes. Regular tapping with intervals of 2–4 days significantly stimulates latex yield on both virgin (never tapped before) and resting (tapped before but left with no tapping for 3 months or above) *Hevea* trees in a similar way, except that the number of tappings required is larger in the former (7–10) than the latter (4–6) to yield at a relatively stable level (Pakianathan et al., 1992; Tang et al., 2010; Qi, 2011). In the latex of virgin *Hevea* trees, expressions of a number of genes involved in latex production are bolstered by the tapping treatment, e.g., *HbSUT3* (Tang et al., 2010), *HbNIN2* (Liu et al., 2015) and a farnesyl diphosphate synthase gene (Adiwilaga and Kush, 1996). Latex transcript and protein profiling of virgin or resting *Hevea* trees for the first few tappings could be beneficial to unraveling the mechanisms of tapping-induced latex production and to identifying the genes or proteins involved.

Since the advent of the cDNA-AFLP transcript-profiling technique in 1996 (Bachem et al., 1996), owing to its advantages of good repeatability, high sensitivity and high throughput, this technique has been successfully applied to various aspects of plant studies, such as plant’s abiotic stress response (Fatemi et al., 2019), plant-microorganism interaction (Xiao et al., 2016), hormone signaling (Akihiro et al., 2006), and plant development (Leymarie et al., 2007). In this study, the latex transcriptome and proteome were compared for the first five tappings in virgin and resting *Hevea* trees, respectively, using a silver staining cDNA-AFLP (Xiao et al., 2009) and two dimensional electrophoresis coupled with mass spectrometry analysis. A large number of differentially expressed (DE) transcripts and proteins were successfully isolated and functionally identified, and the results reveal the importance of sugar metabolism as well as rubber biosynthesis in tapping-activated latex production in *Hevea* trees.

## Results

### Screening of Differentially Expressed Transcripts and Proteins

**FIGURE 1:**
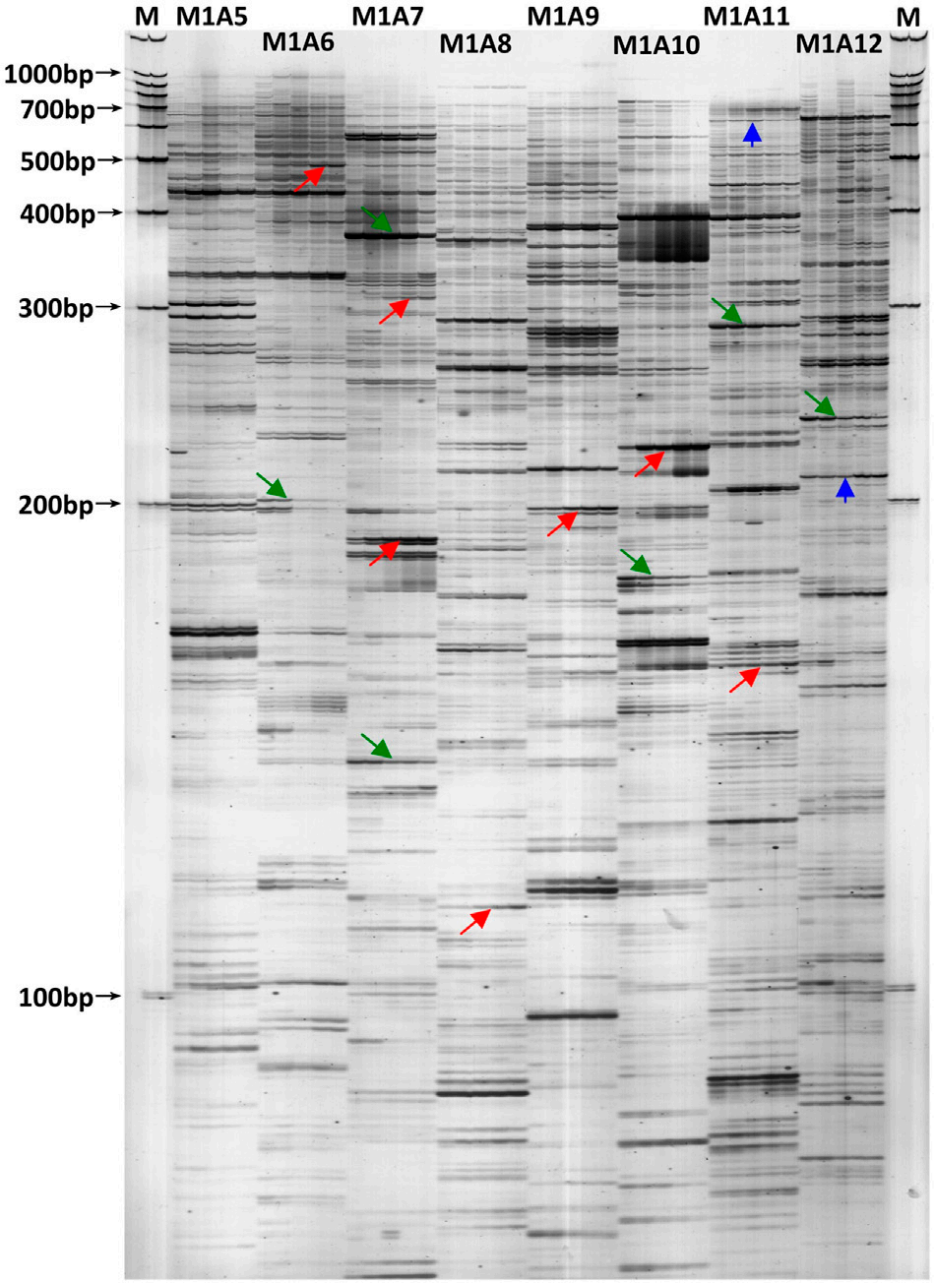
A typical cDNA-AFLP silver-stained polyacrylamide gel revealing transcript profiles in the latex of virgin *Hevea* trees for the first five tappings. M: 100 bp DNA ladder molecular weight standard; M1A5 to M1A12: selective primer combinations. Under each primer pair, the five lanes (from left to right) represent the latex transcript profiles for the first five tappings. The arrows marked in red indicate the DE-TDFs up-regulated by tapping, the green arrows indicate down-regulated, and the blue arrows indicate irregularly-regulated. Please note there exists subtypes for each of the three regulation types.

To extensively identify the differentially expressed transcripts responding to the tapping treatment, all the 128 cDNA-AFLP selective primer pairs of *Apo* I / *Mse* I restriction system (Xiao et al., 2009) were screened in the latex RNA for the first five tappings in virgin *Hevea* trees. On average, about 70 transcript-derived fragments (TDFs) greater than 100 bp were discernable on the silver-stained polyacrylamide gels for each primer pair (Figure 1). Therefore, nearly 9,000 TDFs were profiled for each latex RNA sample. In total, 651 DE-TDFs were successfully cloned and sequenced.

**FIGURE 2:**
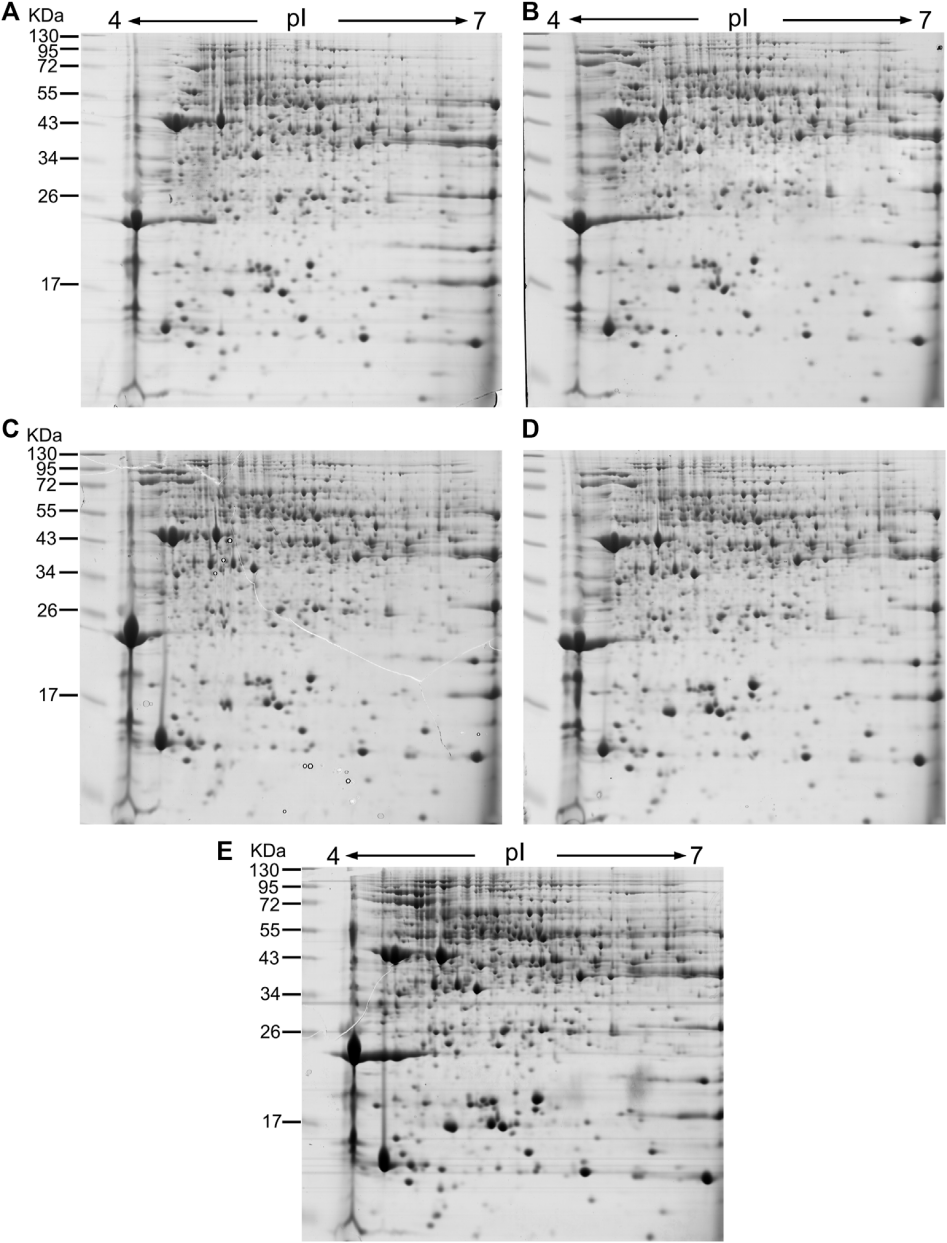
2-DE images of latex C-serum proteins from retapped *Hevea* trees after a four-month resting period. IGP strips of pH4-7 were used in 2-DE, and the fresh latex C-serum samples were analyzed for the first five tappings [**(A)**: first; **(B)**: second; **(C)**: third; **(D)**: fourth; **(E)**: fifth]. kDa: Molecular weight of protein. pI: Protein isoelectric point.

To identify the tapping-responsive proteins, proteomics studies were preformed on re-opened resting *Hevea* trees, the latex production of which is stimulated by tapping in a way similar to virgin *Hevea* trees (Supplementary Figure S1). The resting *Hevea* trees usually yield significantly higher than virgin *Hevea* trees for the first tapping, thus facilitating the conventional proteomics analysis that requires more latex than the virgin *Hevea* trees could produce (Tang et al., 2010; Qi, 2011). For each of the first five tappings in reopened resting *Hevea* trees, 1 mg of latex C-serum proteins were separated on two dimensional electrophoresis (2-DE) gels and stained by Coomassie brilliant blue R250. Most of the protein spots displayed superior resolution with clear background and obvious boundary on the 2-DE gels (Figure 2). On each 2-DE gel, the number of discernable protein spots ranged between 700 and 800 when pH 4–7 IPG strips were used for 1-DE (Figure 2), and between 900 and 1,000 when pH 5–8 IPG strips were used (Supplementary Figure S2). The molecular weights of latex C-serum proteins were concentrated in the range of 15–80 kDa, and the isoelectric points were in pH 4.5–7.5.

Taking the expression level at the first tapping as a reference, the DE-TDFs identified were classified into three types according to their patterns of expression across the five successive tappings: upregulation, downregulation and irregular-regulation (Figure 1). The upregulation type includes three subtypes: 1) increase successively; 2) increase first and then stabilize; 3) increase first and then decrease, but still higher than the first tapping. The downregulation type also includes three subtypes: 1) decrease successively; 2) decrease first and then stabilizes; 3) decrease first and then increase, but still lower than the first tapping. The irregular-regulation includes two subtypes: 1) increase first, reaching a high threshold, and then decrease to a level lower than the first tapping; 2) decrease first, reaching a low threshold, and then increase to a level higher than the first tapping. Accordingly, the DE-protein spots were also classified into the three types according to their expression dynamics across the first five tappings in reoppend resting *Hevea* trees (Figure 2). A protein spot was regarded as a DE one when its abundance varies ≥3-fold between the first tapping and any of the four other tappings. As a result, a total of 117 tapping-responsive DE-protein spots were identified, and picked out from the 2-DE gels for mass spectrometry analysis.

### DE-TDFs and DE-Proteins Annotation and Redundancy Removal

The DE-TDFs were made clean by removing the sequences of vector, primer, and adaptor at both ends and then annotated by Blastx online searching (http://blast.ncbi.nlm.nih.gov/Blast.cgi) against the NCBI non-redundant protein database (nr), with an E-value threshold of <10^−4^ and a score value of >50. According to Blastx search against the *Hevea* latex transcriptome database (Tang et al., 2016), the DE-TDFs belonging to the same transcript and sharing similar expression pattern in the cDNA-AFLP analysis were clustered together, and only the longest TDF was retained. As a result, a total of 505 non-redundant DE-TDFs (GenBank accession nos MZ935745—MZ936248) were obtained, including 217 (43.0%) upregulated (Supplementary Table S1), 180 (35.6%) downregulated (Supplementary Table S2) and 108 (21.4%) irregularly-regulated (Supplementary Table S3). The selected DE-protein spots were identified by a Bruker’s Ultraflex TOF/TOF mass spectrometer. The proteins identified as positive by peptide mass fingerprinting (PMF) analysis were further subjected with 2-4 matched peptides to peptide fragmentation fingerprinting (PFF) investigation. Of the 117 DE-protein spots examined, 89 were identified as positive by the PMF analysis, and 57 were further determined as positive by the PFF analysis. Of the 89 positively identified DE-proteins, 35 (39.3%) were classified as upregulated, 20 (22.5%) as downregulated and 34 (38.2%) as irregularly-regulated (Supplementary Table S4).

Detailed comparison revealed that the DE-protein spots of three small rubber particle proteins (nos. 163, 723 and 730), one tubulin alpha-3 chain-like protein (no. 552) and two latex abundant family proteins (nos. 554 and 628) had their DE-TDF counterparts (M1-A10-2, M12-A6-4, M6-A10-4, M6-A7-1 and M8-A7-2) (Supplementary Tables S1, S2, S4, S5), of which only one pair (protein spot 163 and TDF #M1-A10-2) revealed a consistency of upregulation. The other nine DE-TDFs overlapped in functional annotations with a number of DE- protein spots, belonging to distinct genes within the same gene families (Supplementary Table S5). As reported in studies covering various types of organisms (Taniguchi et al., 2010; Vogel and Marcotte, 2012; Walley et al., 2013), low overlap and poor correlation have been frequently observed between transcriptomic and proteomic data due to several possible explanations. One more plausible explanation for the low overlap revealed here is the detection limit of the proteome technique that is capable of profiling only medium to high expressed proteins (Humphery-Smith et al., 1997) in comparison with the high transcript-detecting sensitivity of the cDNA-AFLP technique (Vuylsteke et al., 2007; Xiao et al., 2009).

### Functional Classification of Non-redundant DE-TDFs and DE-Proteins

According to the results of Blastx search, the 505 non-redundant DE-TDFs were divided into four categories: 1) Proteins with clear functional annotation; 2) Unclassified proteins, the functional annotation of the proteins being multiple; 3) Predicted protein, showing high homology with a predicted protein in the database; 4) No hit, no homologous sequence in the database. Most (366, 72.5%) of these DE-TDFs were homologous to genes with known functions, whereas 30 (5.9%) and 59 (11.7%), respectively, belonged to unclassified proteins and predicted proteins and the remaining 50 (9.9%) with no hit (Table 1). Meanwhile, the 89 positive DE-protein spots were divided into two categories: 1) Proteins with clear functional annotation; 2) Unknown proteins. Most (78, 87.6%) of these DE-protein spots were functionally annotated, whereas the remaining 11 (12.4%) belonged to predicted or hypothetical proteins (Table 1).

**TABLE 1:** Functional categories and statistics of DE-TDFs and DE-protein spots.

Category	No.	Percentage^a^ (%)	Expression pattern^b^ (%)		
			Up	Down	Irregular
DE-TDFs					
Primary metabolism	30	5.9	15 (50.0)	12 (40.0)	3 (10.0)
Energy	20	4.0	8 (40.0)	5 (25.0)	7 (35.0)
Cell growth and division	19	3.8	11 (57.9)	5 (26.3)	3 (15.8)
Transcription and protein synthesis	96	19.0	41 (42.7)	35 (36.5)	20 (20.8)
Protein degradation and storage	34	6.7	18 (52.9)	9 (26.5)	7 (20.6)
Transporters and intracellular transport	45	8.9	20 (44.4)	18 (40.0)	7 (15.6)
Cellular structure	13	2.6	7 (53.8)	6 (46.2)	0
Signal transduction	42	8.3	12 (28.6)	18 (42.8)	12 (28.6)
Stress and defense	46	9.1	23 (50.0)	16 (34.8)	7 (15.2)
Secondary metabolism	11	2.2	8 (72.7)	2 (18.2)	1 (9.1)
Rubber biosynthesis	10	2.0	9 (90.0)	0	1 (10.0)
Unclassified proteins	30	5.9	7 (23.3)	16 (53.4)	7 (23.3)
Predicted proteins	59	11.7	24 (40.7)	20 (33.9)	15 (25.4)
No hit sequence	50	9.9	14 (28.0)	18 (36.0)	18 (36.0)
Total	505	100.0	217 (43.0)	180 (35.6)	108 (21.4)
DE-protein spots					
Primary metabolism and energy	18	20.2	8 (44.4)	5 (27.8)	5 (27.8)
Cell growth, division and structure	12	13.5	4 (33.3)	3 (25.0)	5 (41.7)
Transcription and protein synthesis	9	10.1	5 (55.6)	3 (33.3)	1 (11.1)
Signal transduction	4	4.5	4 (100)	0	0
Stress and defense	23	25.8	4 (17.4)	4 (17.4)	15 (65.2)
Secondary metabolism	4	4.5	3 (75.0)	1 (25.0)	0
Rubber biosynthesis	8	9.0	3 (37.5)	2 (25.0)	3 (37.5)
Unclassified proteins	11	12.4	4 (36.4)	2 (18.1)	5 (45.5)
Total	89	100	35 (39.3)	20 (22.5)	34 (38.2)

a The percentage in each category accounting for the total DE-TDFs and DE-protein spots.

b The number and percentage of DE-TDFs and DE-protein spots distributed in the three expression modes respectively in each category. Up: up-regulated; Down: down-regulated; Irregular: irregularly-regulated.

**FIGURE 3:**
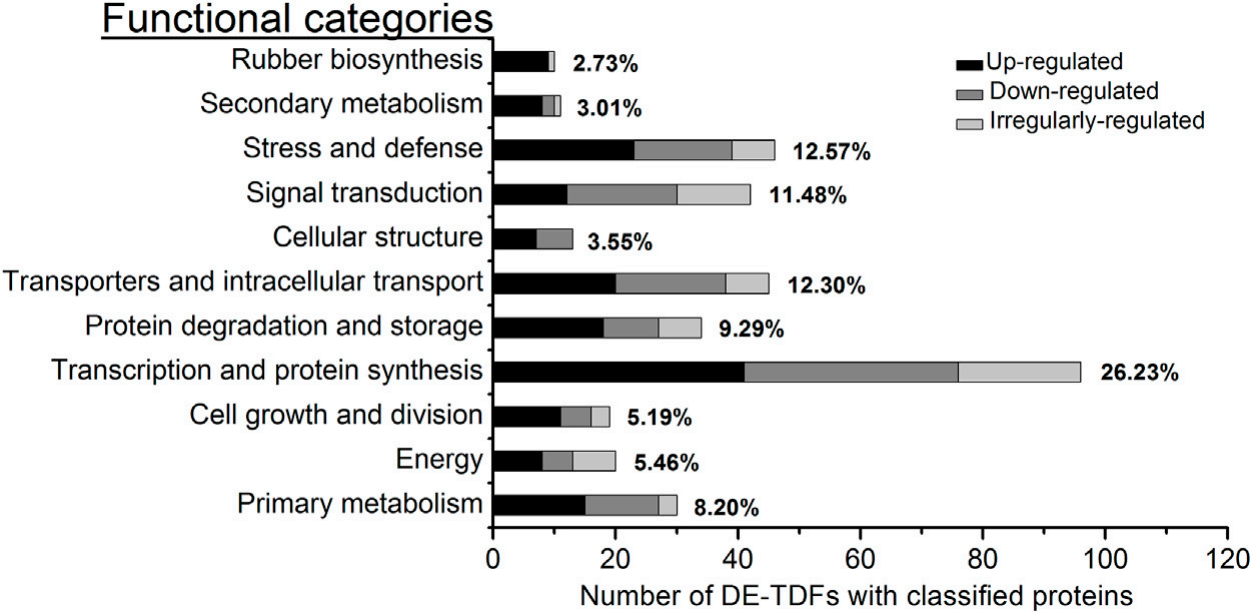
Functional category and percentage of the DE-TDFs with known function. The 366 non-redundant DE-TDFs of annotation proteins with known function were identified in the latex for the first five tappings in rubber trees. These DE-TDFs were classified into 11 functional categories. In each category, the percentage covering the total know functional DE-TDFs was placed at its right whereas the three types of expression were shown in differing gray bars.

With reference to the functional categories of plant genes defined by Bevan et al. (1998), the 366 DE-TDFs and 78 DE-proteins with known functions were classified, respectively, into 11 and 7 functional categories, among which a new category, rubber biosynthesis, was singled out from “secondary metabolism” (Figure 3; Table 1). Of these DE-TDFs, five categories including cell growth and division, protein degradation and storage, cellular structure, secondary metabolism, and rubber biosynthesis had a higher portion of upregulated DE-TDFs than that of the down- and irregularly-regulated DE-TDFs together (Table 1). Noteworthily, nine of the ten DE-TDFs implicated in rubber biosynthesis were upregulated, and the remaining one is irregularly-regulated. Of the 89 DE-protein spots, the number of upregulated (35) was significantly larger than the downregulated (20), but similar to the irregular-regulated (34). Expression patterns of the DE-protein spots varied in most functional categories, whereas all four in the signal transduction category were upregulated. It is worth noting that there were many cases that more than one protein spots corresponded to an identical gene, e.g., six spots (nos. 33, 229, 516, 534, 539, and 591) for an enolase, five (nos. 149, 163, 723, 730, and 858) for a small rubber particle protein, and five (nos. 688, 778, 779, 789, 797, and 803) for a heat shock protein (Supplementary Table S4), indicating extensive posttranslational modifications in *Hevea* latex proteins.

### Validation of Expression Pattern by sqRT-PCR

To determine the reliability of the cDNA-AFLP results, 80 DE-TDFs, covering >10% of the DE-TDFs we identified were randomly selected from each functional category and subjected to semi-quantitative reverse transcription PCR (sqRT-PCR) analysis using specific primers for TDFs with *18S rRNA* as the reference gene. The ImageJ software was used to quantify the sqRT-PCR amplicons fractionated in agarose gel electrophoresis, with the value in the first tapping sample taken as 1.0 (Table 2). About 84% (67 TDFs) showed expression patterns consistent with their cDNA-AFLP gel profiles, indicating the high reliability of the cDNA-AFLP screening.

**TABLE 2:** Semi-quantitative RT-PCR validation of DE-TDFs for the profiles in virgin *Hevea* trees.

DE-TDFs	cDNA-AFLP	sqRT-PCR^a^	Consistency^b^	DE-TDFs	cDNA-AFLP	sqRT-PCR^a^	Consistency^b^
M1-A5-1	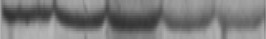	1.00, 1.09, .91, .80, .76	+	M9-A9-1	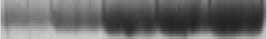	1.00, 1.42, 2.48, 3.18, 4.93	+
M1-A6-2	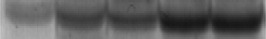	1.00, 1.16, .89, 2.85, 3.23	+	M9-A10-1	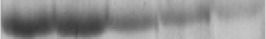	1.00, 1.13, .78, 0.61, .56	+
M1-A6-3	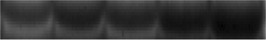	1.00, 1.10, 1.19, 1.40, 1.32	+	M9-A10-2	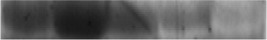	1.00, 1.69, 1.15, .88, .59	+
M1-A7-1	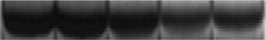	1.00, 0.94, 0.47, 0.76, 0.51	+	M9-A11-1	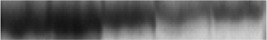	1.00, 1.02, .88, .61, 0.44	+
M1-A7-2	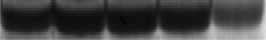	1.00, 1.00, .94, .85, 0.18	+	M9-A11-2	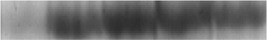	1.00, 1.11, 1.26, 1.33, 1.08	+
M1-A7-3	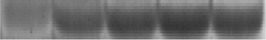	1.00, 1.13, 1.27, 1.52, 1.82	+	M9-A11-3	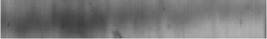	1.00, 1.02, .84, .75, .66	+
M1-A7-4	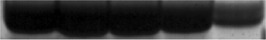	1.00, .94, .65, .73, .55	+	M9-A12-1	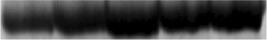	1.00, 1.08, 1.22, 1.47, 1.57	+
M1-A7-5	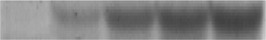	1.00, 1.13, 1.24, 1.26, 1.35	+	M10-A8-2	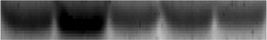	1.00, 1.18, .74, .71, .65	+
M1-A8-1	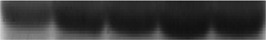	1.00, 1.29, 1.31, 1.55, 1.71	+	M10-A9-1	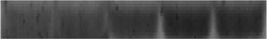	No band	−
M1-A8-2	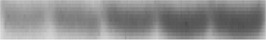	No band	−	M10-A9-3	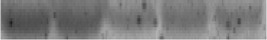	1.00, .96, .79, .77, .76	+
M1-A9-1	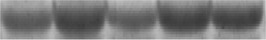	1.00, 1.02, 1.06, 1.24, 1.23	+	M10-A10-1	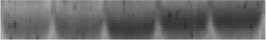	1.00, 1.13, 1.42, 1.70, 2.05	+
M1-A9-2	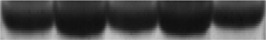	1.00, 1.11, .86, .92, 1.03	−	M10-A11-1	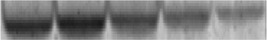	1.00, 1.11, .62, .51, .33	+
M1-A9-3	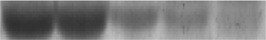	1.00, .91, 1.04, 1.23, 1.27	−	M10-A11-2	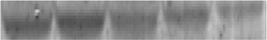	1.00, .78, .48, .40, .20	+
M1-A10-1	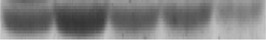	1.00, .76, .65, .40, .36	+	M10-A12-1	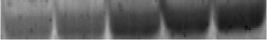	1.00, 1.56, 1.69, 2.45, 2.47	+
M1-A10-2	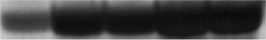	1.00, 1.44, 2.91, 3.42, 3.81	+	M10-A12-2	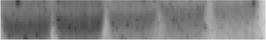	1.00, 1.12, .62, 0.59, .55	+
M1-A10-3	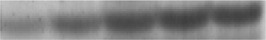	1.00, 1.03, 1.27, 1.40, 2.03	+	M11-A7-1	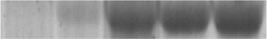	1.00, 1.14, 1.57, 1.59, 1.80	+
M1-A11-1	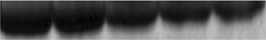	1.00, .95, .75, .75, .74	+	M11-A8-1	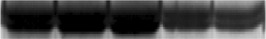	1.00, .88, .58, .55, .46	+
M1-A11-2	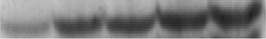	No band	−	M11-A8-2	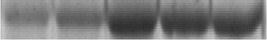	1.00, 1.32, 1.69, 2.25, 2.20	+
M1-A11-3	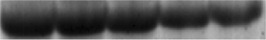	1.00, .97, .81, .81, .78	+	M11-A9-1	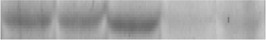	1.00, .89, .67, .65, .57	+
M1-A11-4	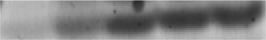	1.00, .93, .87, .94, 1.00	−	M11-A9-2	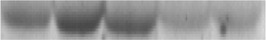	1.00, 1.07, .77, 0.77, 0.66	+
M1-A12-2	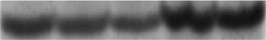	1.00, 1.00, .82, .95, .96	+	M11-A10-1	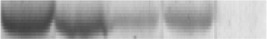	1.00, 1.18, 1.15, 1.16, 1.19	−
M2-A5-1	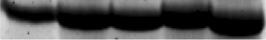	1.00, 1.34, 1.64, 1.91, 1.97	+	M15-A5-1	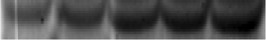	1.00, 1.25, 1.61, 1.77, 2.27	+
M2-A5-2	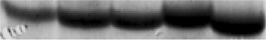	1.00, 1.04, 1.07, 1.12, 1.13	+	M15-A8-1	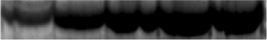	1.00, 1.46, 1.85, 2.03, 2.48	+
M2-A6-2	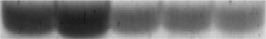	1.00, 1.12, .86, .66, 0.65	+	M15-A8-4	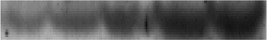	1.00, 1.03, 1.18, 1.36, 1.67	+
M2-A7-1	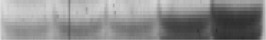	1.00, 1.01, 1.18, 1.57, 2.22	+	M15-A9-1	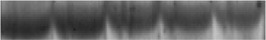	1.00, .78, .51, .49, .49	+
M2-A7-3	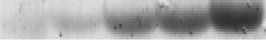	1.00, 1.38, 2.51, 3.04, 3.78	+	M15-A10-1	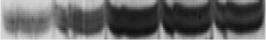	1.00, 2.09, 1.61, 1.49, 1.19	−
M2-A7-5	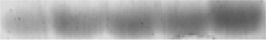	1.00, 1.04, 1.23, 1.29, 1.63	+	M15-A10-3	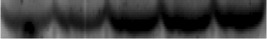	1.00, 1.06, 1.27, 1.45, 1.66	+
M2-A8-1	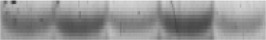	1.00, 1.12, 1.22, 1.46, 1.31	+	M15-A10-4	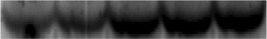	1.00, .98, .61, .64, .40	−
M2-A8-2	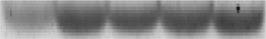	1.00, .99, .94, 1.18, 1.08	−	M16-A5-1	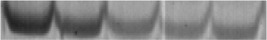	1.00, .80, .58, 0.55, 0.51	+
M2-A9-1	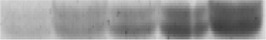	1.00, 1.70, 1.85, 2.06, 2.11	+	M16-A6-1	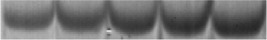	1.00, 1.06, 1.12, 1.51, 1.72	+
M2-A9-3	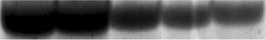	1.00, .97, .86, .83, 0.81	+	M16-A7-1	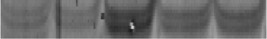	1.00, 1.12, 1.07, 1.41, 2.23	−
M2-A10-1	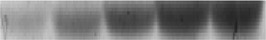	1.00, 1.56, 2.40, 3.34, 3.94	+	M16-A8-1	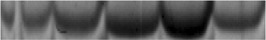	1.00, 1.30, 1.41, 1.69, 1.20	+
M2-A10-2	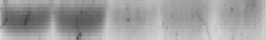	1.00, .88, .47, .68, 0.41	+	M16-A9-1	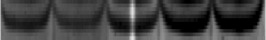	1.00, .56, .76, 1.03, 1.22	−
M2-A10-3	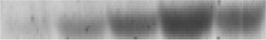	1.00, .99, 1.12, 1.33, 1.22	+	M16-A9-2	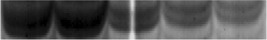	1.00, .99, .85, .68, .49	+
M3-A7-1	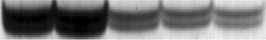	1.00, 1.06, .69, 0.68, 0.41	+	M16-A10-1	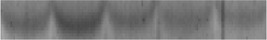	1.00, .94, .71, .65, 0.44	+
M3-A10-3	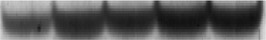	1.00, 1.60, 2.12, 2.51, 2.79	+	M16-A10-2	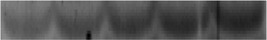	1.00, 1.07, 1.23, 1.54, 1.88	+
M3-A11-1	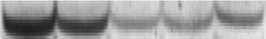	1.00, .74, .37, .45, 0.50	+	M16-A11-1	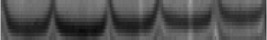	1.00, .85, .70, .62, .61	+
M9-A6-1	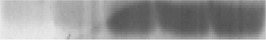	1.00, 1.07, 1.09, 1.30, 1.65	+	M16-A11-2	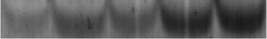	1.00, 1.12, 1.14, 1.22, 1.15	+
M9-A7-1	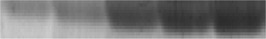	1.00, 1.39, 1.71, 2.30, 2.85	+	M16-A12-1	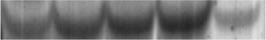	1.00, .81, 1.10, 1.22, 2.04	−
M9-A8-1	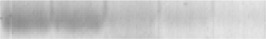	1.00, .95, .63, .61, .61	+	M16-A12-3	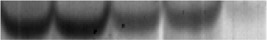	1.00, .83, .52, .50, .47	+

a With the scanned intensity for the TDF band of the first tapping set as 1.00, the relative expression of DE-TDFs were calculated for the first five tappings.

b “+” means that the result of sqRT-PCR is consistent with that of cDNA-AFLP analysis; “−” indicates inconsistency between the two analysis.

### qRT-PCR Analysis of Latex Regeneration-Related DE-TDFs

**FIGURE 4:**
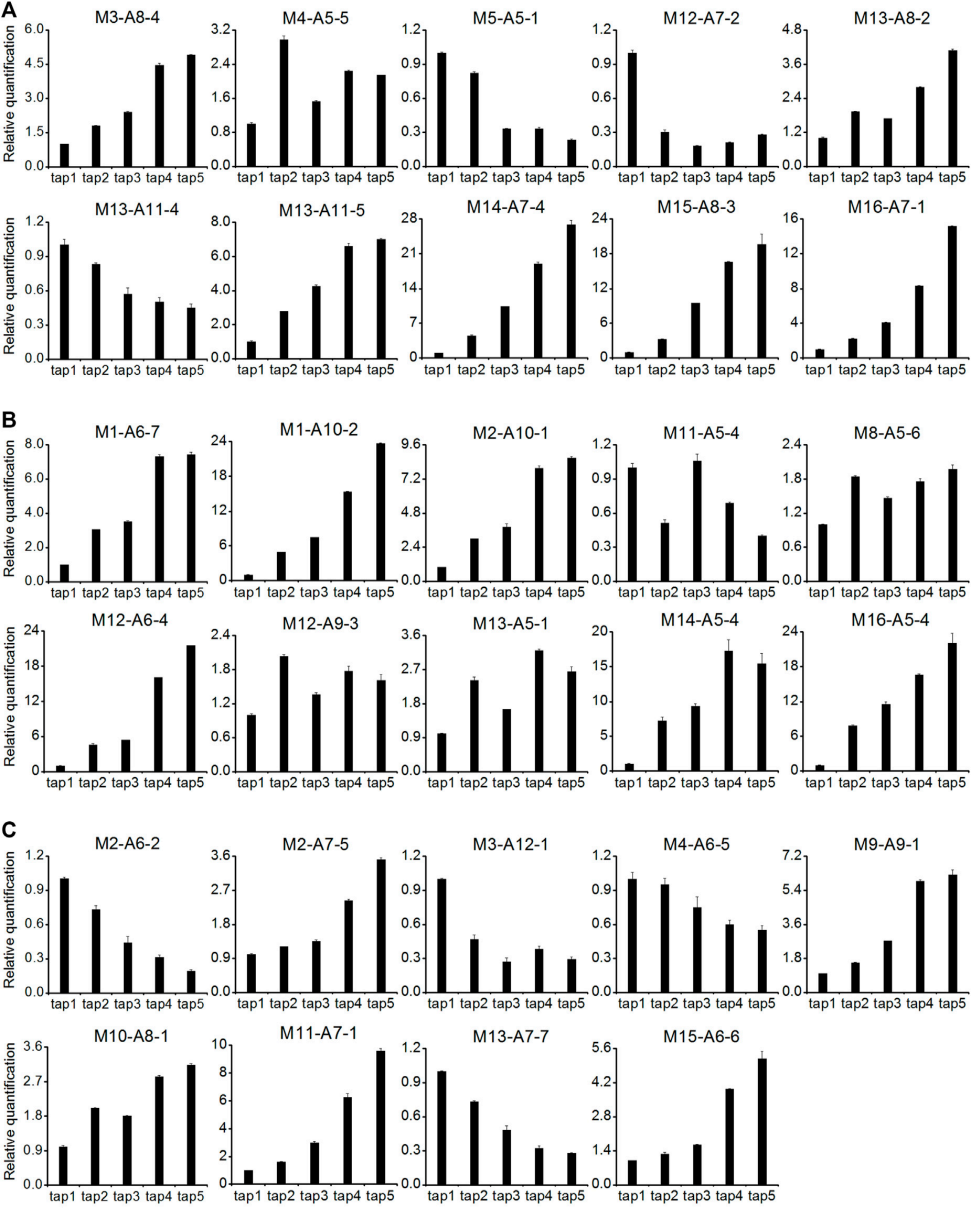
qRT-PCR analysis for expression of latex regeneration genes in the latex of the first five tappings. **(A)**: primary metabolism pathway (ten DE-TDFs); **(B)**: rubber biosynthesis and regulation pathway (ten DE-TDFs); **(C)**: transporters and intracellular transport pathway (nine DE-TDFs). Except for M3-A8-4, M11-A5-4 and M16-A7-1, 26 of the 29 DE-TDFs shows the results consistent with their original cDNA-AFLP expression patterns and were presented here. All data were normalized to the expression level of *HbYLS8* gene. Values are averages ± STDEV of three technical replicates. The latex samples of three individual trees were analyzed with similar patterns of expression, and one representative was shown.

A total of 29 DE-TDFs implicated in latex-regeneration were investigated by quantitative RT-PCR (qRT-PCR) analysis for their expression patterns across the five successive tappings. About 90% of the qRT-PCR results were consistent with their original cDNA-AFLP expression profiles (Figure 4; Table 3). The genes of these DE-TDFs are putatively involved in the pathways of primary metabolism, rubber biosynthesis and regulation, transporters, and intracellular transport. Of the ten rubber biosynthesis pathway DE-TDFs, nine revealed qRT-PCR patterns similar to their cDNA-AFLP results (Figure 4; Table 3).

**TABLE 3:** Information of latex regeneration related DE-TDFs for qRT-PCR analysis.

DE-TDFs	GenBank blastx annotation	Model	Consistency^a^
Primary metabolism (10)			
M3-A8-4	pyruvate dehydrogenase, putative	Irregular	−
M4-A5-5	NADP-dependent glyceraldehyde-3-phosphate dehydrogenase, putative	Irregular	+
M5-A5-1	G6PD1 (glucose-6-phosphate dehydrogenase)	Irregular	+
M12-A7-2	Phosphofructokinase, putative	Irregular	+
M13-A8-2	pyruvate dehydrogenase, putative	Up	+
M13-A11-4	mitochondrial pyruvate dehydrogenase kinase isoform 2	Down	+
M13-A11-5	pyruvate kinase, putative	Up	+
M14-A7-4	Fructokinase, putative	Up	+
M15-A8-3	glyceraldehyde-3-phosphate dehydrogenase	Up	+
M16-A7-1	neutral/alkaline invertase	Up	−
Rubber biosynthesis and regulation (10)			
M1-A6-7	cis-prenyltransferase	Up	+
M1-A10-2	small rubber particle protein	Up	+
M2-A10-1	rubber elongation factor	Up	+
M8-A5-6	inorganic pyrophosphatase, putative	Irregular	+
M11-A5-4	farnesyl diphosphate synthase	Up	-
M12-A6-4	small rubber particle protein	Up	+
M12-A9-3	3-hydroxy-3-methylglutaryl-coenzyme A reductase 2	Up	+
M13-A5-1	rubber elongation factor	Up	+
M14-A5-4	rubber elongation factor	Up	+
M16-A5-4	hydroxymethylglutaryl coenzyme A synthase	Up	+
Transporters and intracellular transport (9)			
M2-A6-2	AGD13 (ARF-GAP domain 13); ARF GTPase activator/zinc ion binding	Irregular	+
M2-A7-5	vacuolar ATP synthase subunit G plant, putative	Up	+
M3-A12-1	RAB6A; GTP binding/protein binding	Irregular	+
M4-A6-5	small Ras-like GTP-binding protein	Down	+
M9-A9-1	AtRABA1f (Arabidopsis Rab GTPase homolog A1f); GTP binding	Up	+
M10-A8-1	sugar transporter, putative	Up	+
M11-A7-1	vacuolar ATP synthase proteolipid subunit 1, 2, 3, putative	Up	+
M13-A7-7	prenylated Rab acceptor protein, putative	Down	+
M15-A6-6	AtRABH1e (Arabidopsis Rab GTPase homolog H1e); GTP binding	Up	+

a “+” means that the expression pattern of DE-TDFs by qRT-PCR are consistent with the cDNA-AFLP gel profiles, and “−” means that the expression pattern are inconsistent.

## Discussion

### Functional Categories With Reference to Tapping-Stimulated Latex Production

Tapping can stimulate latex production, especially in virgin and reopened resting *Hevea* trees (Pakianathan et al., 1992; Tang et al., 2010; Qi, 2011). A number of early studies have shown that the first few tappings greatly stimulate the metabolism of laticifers, accompanied by the enhanced expression of several specific genes involved in latex production (Dennis et al., 1989; Adiwilaga and Kush, 1996; Gohet, 1996; Priya et al., 2007). The latex flows out of laticifers after tapping, and in order to compensate for the loss of cytoplasm (latex) and maintain the balance of intracellular metabolism, the laticifers require large amounts of RNA and proteins to be synthesized before the next tapping. Of the 366 DE-TDFs identified in the latex with known functions (Table 1), 26.2% were classified into the functional category of transcription and protein synthesis (Figure 3), representing the largest category, 42.7% of which were upregulated by the tapping treatment. These results indicated that tapping significantly affects the ways of laticifers to synthesize RNA and proteins, providing a prerequisite for multiple subsequent biological responses to the tapping treatment. Of the 78 DE-protein spots identified in the latex with known functions, 29.5% were classified into the functional category of stress and defense (Table 1) and represented the largest category, corresponding well to the defense functions of *Hevea* laticifers (Sharples, 1918; Chow et al., 2007). Laticifers are believed to be a defense system for *Hevea* trees to cope with biotic and abiotic stresses, and the latex exuded after bark wounding has been found to play roles in resisting pathogen infection, insect feeding and abiotic stress (Chow et al., 2007). The tapping itself is a kind of abiotic stress upon *Hevea* trees. Consistent with the proteome study, “stress and defense” also accounted for a large portion of the functional DE-TDFs, ranking the second place among the DE-TDF functional categories (Figure 3; Table 1; Supplementary Table S4). The harvesting stress response has been suggested to be one of the key factors affecting latex production and rubber productivity in *Hevea* trees (Pirrello et al., 2014).

The category of transporters and intracellular transport was the third largest among the 11 functional categories, accounting for 12.3% of the total functional DE-TDFs (Figure 3). This agrees well with the sink effect caused by the large loss of latex after tapping. The process of regenerating the expelled latex involves the synthesis, transport, loading and subcellular localization of a large number of organelles, proteins, nucleic acids, sugars, etc., all of which require the active involvement of transporters and intracellular transport-related proteins (d’Auzac et al., 1997; Tungngoen et al., 2011). DE-TDFs involved in signal transduction were also highly represented, accounting for a proportion of 8.3% for the total DE-TDFs (Table 1), and all four DE-proteins involved in signal transduction were upregulated (Table 1; Supplementary Table S4). A variety of signaling pathways within *Hevea* laticifers, including ethylene, jasmonic acid and wound signaling, have been reported to be extensively participate in latex regeneration and regulation (Dennis et al., 1989; Hao et al., 2004; Duan et al., 2010; Lacote et al., 2010; Pirrello et al., 2014; Tang et al., 2016). The proportions for the two categories, protein degradation and storage and primary metabolism were also high, covering, respectively, 9.3% and 8.2% of the total functional DE-TDFs (Figure 3; Table 1). Their high representation suggested that with the progress of tapping, in order to meet the balance of supply and demand of all substances in latex regeneration, protein turnover rate becomes faster and primary metabolism gets active. In a word, these results indicated that the latex production induced by tapping involves a complex multi-gene regulatory network, as well as multiple physiological and biochemical response processes.

### Sugar Metabolism and Rubber Biosynthesis in Tapping-Stimulated Latex Production

In regularly tapped *Hevea* trees, the main metabolic activity of the laticifers is latex regeneration, which centers on the biosynthesis of NR that consists of about 90% of the latex dry weight (d’Auzac et al., 1997). Sucrose is the precursor material for rubber biosynthesis in laticifers, and provides the carbon skeleton and energy required for latex regeneration (Tupý, 1989; Duangngam et al., 2020). In *Hevea* trees that are tapped at two-four days of intervals, a tree produces dozens to hundreds of milliliters of fresh latex, and the removed latex could be effectively recovered before the next tapping (d’Auzac et al., 1997; Tang et al., 2010). Therefore, the laticifers represent an active carbon sink, and the effective supply of sucrose is a key factor determining latex production (Tupý, 1985; Chantuma et al., 2009). In this study, the genes of a sucrose transporter (M10-A9-1) and a sugar transporter (M10-A8-1) were among the DE-TDFs identified, both of which were significantly upregulated with the increase of tappings (Supplementary Table S1). Interestingly, the sucrose transporter (M10-A9-1) identified here is the HbSUT3 we previously reported to be critical in sucrose uptake into laticifers and *Hevea* rubber production (Tang et al., 2010). The upregulation of these two transporters indicated an active involvement of sucrose and sugar transport in tapping-stimulated latex production. Sucrose catabolism and the following pathways including glycolysis, tricarboxylic acid cycle and pentose phosphate pathway provide essential components, i.e., the carbon skeleton (acetyl CoA), the reducing power (NADPH) and the energy (ATP) for the final rubber biosynthesis pathway (Tupý, 1989; d’Auzac et al., 1997). Therefore, sugar metabolism becomes one of the core metabolic pathways contributing to latex production in *Hevea* (Tupý, 1989; d’Auzac et al., 1997; Silpi et al., 2007). This study identified multiple DE-TDFs involved in sucrose cleavage and the three above mentioned sugar metabolism pathways (Table 3; Supplementary Tables S1–S3), including neutral/alkaline invertase (M16-A7-1), fructokinase (M14-A7-4), phosphofructokinase (M12-A7-2), glyceraldehyde 3-phosphate dehydrogenase (M15-A8-3), pyruvate kinase (M13-A11-5), pyruvate dehydrogenase (M13-A8-2), and glucose-6-phosphate dehydrogenase (M5-A5-1), etc. Most of these DE-TDFs were upregulated in the latex for the first few tappings (Figure 3; Supplementary Table S1). It is worth noting that the upregulated DE-TDF (M16-A7-1) as identified by both cDNA-AFLP (Supplementary Table S1) and qRT-PCR (Figure 4) turned out to be *HbNIN2*, the neutral/alkaline invertase that is responsible for sucrose catabolism in *Hevea* laticifers (Liu et al., 2015). Here, the proteomic research also backed up the importance of sugar metabolism in tapping-stimulated latex production in reopened resting *Hevea* trees although a low overlap was observed among the DE-genes and DE-proteins we identified this study. Among the 18 DE-protein spots identified in category of primary metabolism and energy, 13 were involved in sugar metabolism (Supplementary Table S4). Notably, nine spots (nos. 356, 372, 593, 33, 229, 516, 535, 539, and 591) were implicated in the glycolytic pathway. By contrast, no DE-protein spots were in tricarboxylic acid (TCA) cycle, collaborating the proposition of the relative importance of glycolysis versus the TCA cycle in sugar degradation in hypoxic *Hevea* latex (d’Auzac and Jacob, 1969; Tupý and Resing, 1969) and thus in tapping-stimulated latex production.

There are 20 gene families directly involved in the NR biosynthesis and termed as rubber biosynthesis (RB) genes (Tang et al., 2016; Chow et al., 2007). In this study, a total of nine DE-TDFs involving six such families were identified, including cis-prenyltransferase (M1-A6-7), hydroxymethylglutaryl coenzyme A synthase (M16-A5-4), 3-hydroxy-3-methylglutaryl-coenzyme A reductase (M12-A9-3), farnesyl diphosphate synthase (M11-A5-4), REF/SRPP proteins (M2-A10-1, M13-A5-1, M14-A5-4, M1-A10-2, M12-A6-4) (Table 3). Among the nine DE-TDFs, eight were demonstrated by qRT-PCR to be upregulated with the tappings (Figure 4). In addition, a DE-TDF (M8-A5-6) annotated as inorganic pyrophosphatase, a vacuolar type of which being located on rubber particles and essential for IPP incorporation into elongating rubber hydrocarbon molecules (Zeng et al., 2009), was also bolstered by the tapping treatment (Figure 4). The tapping treatment also changes the expression of RB genes at the protein levels in reopened resting *Hevea* latex. A total of eight relevant DE-protein spots were identified, corresponding to REF/SRPP proteins and an acetyl-CoA C-acetyltransferase (Supplementary Table S4).

### Strength and Weakness of the cDNA -AFLP Technique

The cDNA-AFLP technique has been widely applied in various eukaryotes including the *Hevea* tree for transcript profiling due to its advantages of stringency, reproducibility, cost-effectiveness, genome-wide coverage and the ability to distinguish among highly homologous genes (Ko et al., 2003; Vuylsteke et al., 2007; Ganeshan et al., 2011; Tang et al., 2013; Xiao et al., 2016). In this study, as determined by sqRT-PCR and qRT-PCR, about 84% and 90%, respectively, of the selected DE-TDFs were verified for their cDNA-AFLP profiles (Figure 4; Tables 2, 3), reflecting a high reliability of this technique in screening tapping-responsive DE-TDFs in *Hevea* latex. According to a previous *in-silico* estimation (Xiao et al., 2009), about 84% of the genes expressed in *Hevea* latex could be visualized using the silver-staining cDNA-AFLP technique with the restriction enzyme pair of *Apo* I and *Mse* I exploited here. The sucrose transporter HbSUT3 (Tang et al., 2010) and the neutral/alkaline invertase HbNIN2 (Liu et al., 2015) that have been reported to be upregulated in the latex of virgin *Hevea* trees by the tapping treatment were among the DE-TDFs identified in this study (Figure 4; Table 3; Supplementary Table S1), demonstrating a high transcript coverage of this technique. However, compared with the currently popularly used next generation RNA-sequencing technique that relies on expensive DNA sequencers and specialized bioinformatics, the cDNA-AFLP is labor-intensive. Nevertheless, the cDNA-AFLP technique still have its niche among the various transcript profiling techniques, and can be readily established in a mediocrely equipped and stringently funded lab to fulfill its customized transcript profiling task.

## Conclusion

An integrative transcriptome and proteome analysis was conducted to identify important regulators and pathways participated in tapping stimulated latex production in *Hevea* trees. A total of 505 tapping-responsive DE-TDFs and 89 DE-proteins were identified in the rubber-producing laticifers of *Hevea* trees (virgin or reopened). The low overlap between the DE-TDFs and DE-protein spots identified is indicative of posttranscriptional regulation and the strong complementarities of transcriptome and proteome analysis. According to the 366 DE-TDFs and 78 DE-proteins with functional annotations, the tapping treatment brought about extensive physiological and molecular changes in laticifers. The integration of these changes, especially those of sugar metabolism and rubber biosynthesis, upgraded the mediocre level of laticifer metabolism in virgin or reopened trees to a high dynamic equilibrium of latex regeneration in regularly tapped trees. Further integrative studies will benefit a deeper insight into the exact relationships (synergy or antagonism) among the vast number of biological pathways implicated in tapping-stimulated latex production.

## Materials and Methods

### Plant Materials

*Hevea* trees of Reyan7-33-97 clone for cDNA-ALFP transcript profiling (virgin trees) and 2-DE protein profiling (resting trees) analysis, were all planted in the experimental field of Chinese Academy of Tropical Agricultural Sciences (Danzhou, Hainan). i.e., removing a slice of trunk bark by a special knife, with a half spiral tapping system, every 3 days, and with no ethylene stimulation (Tang et al., 2010). The virgin trees were planted for eight years and first subjected to the tapping. The resting trees (after four months of no tapping) were planted for 10 years, and re-tapped for the third year. Because the virgin rubber trees produced little latex in the first tapping that could not satisfy the requirement of C-serum preparation for 2-DE analysis as described below, re-tapped resting trees were therefore exploited. Such *Hevea* trees produce much more latex than virgin trees in the first tapping and in the subsequent several tappings the latex production increases in a pattern similar to that observed in virgin trees albeit not striking.

### Extraction of Latex Total RNA

Three batches of five rubber trees attaining the tapping standard (trunk girth >= 50 cm at 1 m above the ground) were selected for latex collection and RNA extraction. Twenty seconds after tapping, about 5 ml of latex was allowed to flow into a centrifuge tube containing 5 ml 2×RNA extraction buffer (.3 M LiCl, 10 mM EDTA, 10% SDS, 100 mM Tris-HCl, pH8.0). The collected latex was placed in ice box and brought to laboratory for RNA extraction as described in Tang et al. (2007). Electrophoresis on a 1.5% formaldehyde denaturing agarose gel was used to detect the integrity of RNA samples. RNA samples from each batch of rubber trees for the five tappings were used as one biological replicate.

### cDNA-AFLP Analysis

A total of 50 μg latex total RNA taken from each of the five tapping samples was subjected to cDNA-AFLP analysis. The detailed manipulations were conducted according to the silver-staining cDNA-AFLP procedure which we previously established suitable for *Hevea* latex transcriptome profiling (Xiao et al., 2009). Briefly, the synthesized double-stranded cDNA was cut by the restriction enzymes of *Apo* I and *Mse* I (Thermo Fisher Scientific, Vilnius, Lithuania), and ligated with adaptors. The ligation product, termed primary template, was used directly for pre-amplification. The pre-amplification product, termed secondary template, was then used for selective amplification. All the 128 possible selective primer combinations with 8 *Apo* I primers and 16 *Mse* I primers as reported previously (Xiao et al., 2009) were applied in screening DE-TDFs affected by the tapping treatment in mature virgin *Hevea* trees.

### DE-TDFs Extraction and Amplification

With the expression level of the TDFs at the first tapping as a reference, the DE-TDFs were identified for the tapping treatment and isolated from the polyacrylamide gel. The DE-TDFs was scraped from the polyacrylamide gel with a surgical blade, put into a sterile PCR tube containing 30 μl .1× TE buffer (10 mM Tris-HCl, 1 mM EDTA, pH8.0), heated at 95°C for 15 min, kept overnight at 4°C, and centrifuged at 10,000 g for 5 min to collect the dissolved DNA solution for PCR amplification. A total of 25 μl PCR reaction mixture includes: 2 μl DNA solution, 2.5 μl 10× PCR buffer (plus Mg^2+^), 1.2 μl 2.5 mM dNTPs Mix, .5 μl 50 ng/μl *Mse*Ⅰselective primer, .5 μl 50 ng/μl *Apo*Ⅰselective primer, .2 μl 5U/μl *Taq* DNA Polymerase (Takara, Dalian, China) and 18.1 μl ddH_2_O. The PCR amplification procedure is the same as the pre-amplification in cDNA-AFLP analysis (Xiao et al., 2009). PCR products were fractionated by 1.2% agarose gel electrophoresis, and the target band was sliced and purified using AxyPrep^TM^ DNA Gel Extraction Kit (AxyGen, shanghai, China).

### DE-TDFs Cloning and Sequencing

The purified DE-TDFs were ligated with the T-vectors using the pMD18-T Vector Kit (Takara, Dalian, China) in accordance with the manufacture’s manual. The ligation mixture was used to transform *E. coli* JM109 competent cells and the transformants were sent to BGI Genomics Co., Ltd. (Shenzhen, China) for sequencing.

### Semi-Quantitative Reverse Transcription PCR

The first strand of cDNA was synthesized by reverse transcriptase kit (RevertAid^TM^ First Strand cDNA Synthesis Kit-K1622, Thermo Fisher Scientific, Vilnius, Lithuania), and then diluted ten times as the template for sqRT-PCR with *18S rRNA* used as the reference (Xiao et al., 2009). The annealing temperatures for the PCR amplification were set according to the primers designed for each specific gene. The PCR cycles were controlled to keep the amplification under the plateau phase. All of the amplified fragments were cloned, and were sequenced for target confirmation. The amount of cDNA samples used in sqRT-PCR was adjusted to be the same for the five tappings based on the level of *18S rRNA* expression, because it is conserved for sqRT-PCR in most of the plant species (Morris and Davila, 1996).

### Quantitative RT-PCR

The expression pattern of candidate genes was detected by qRT-PCR. The first strand of cDNA was diluted 20 times as the template for qRT-PCR with *HbYLS8* as the reference gene which was recommended in our previous study as a suitable internal standard gene in qRT-PCR analysis for the tapping treatment in rubber tree (Li et al., 2011). The PCR reaction mixture includes: 2 μl template, .3 μl each for 10 μM forward and reverse primers, 10 μl 2×SYBR^®^ Premix Ex *Taq* ^TM^Ⅱ (Takara, Dalian, China) and 7.4 μl ddH_2_O. Roche’s LightCycler 2.0 system was used for qRT-PCR analysis with the program as follows: 95°C 30 s; 94°C 5 s, 60°C 20 s, 72°C 20 s, 45cycles. Three technical replicates were analyzed for each of the three biological samples. All the cycle threshold (Ct) values from one gene were determined at the same threshold fluorescence value of .2 using the 2^−ΔΔCT^ method (Livak and Schmittgen, 2001). The primers of target and reference genes were listed in Supplementary Table S6. Student’s *t*-test was performed using Statistical analysis was performed using Student’s t-test.

### Extraction and Dissolution of Latex C-Serum Proteins

Three batches of five rubber trees were selected for latex collection and protein extraction. After tapping, sixty drops of latex were discarded and ∼30 ml latex per tree was then collected in a 50 ml centrifuge tube placed in an ice bath. The collected latex was immediately taken back to the laboratory, and centrifuged at 22, 000 g 4°C for 2 h to acquire the middle aqueous layer, latex cytosol (C-serum). The C-serum proteins were extracted by TCA/acetone-diethyl method (Saravanan and Rose, 2004), freeze-dried, and stored at −80°C. Protein dissolution was performed by incubating for 1 h at 4°C with ultrasonic treatment for 5 min (2s on, 3s off, 15W), with a proportion of 1 mg freeze-dried protein powder added into 10 μl lysis buffer (7M urea, 2M thiourea, 4% CHAPS, 2 mM TBP, 65 mM DTT, .2% w/v IPG buffer). The insoluble precipitate was removed by centrifugation at 20°C 15, 000 g for 10 min, and the supernatant was used for protein separation by two-dimensional electrophoresis (2-DE).

### Protein Separation by 2-DE

The protein content was determined according to Bradford (1976). For each of the first five tappings conducted on re-tapped resting *Hevea* trees, 1 mg of latex C-serum proteins were dissolved in hydration loading buffer [7M urea, 4% CHAPS, 65 mM DTT, .2% Bio-Lyte pH3/10 or pH5/8 ampholyte, .002% (W/V) bromophenol blue] to a final volume of 300 μl. The protein mixture was centrifuged at 20°C 15, 000 g for 20 min to remove bubbles and undissolved precipitates, and then loaded into the IPG strips (Bio-Rad, USA) as described in Sanchez et al. (1997). The strips were then subjected to isoelectric focusing and SDS-PAGE, in an Ettan IPGphor and DALT system according to the manufacturer’s 2-DE manual (GE Healthcare). Three biological replicates for each tapping were conducted.

### Gel Staining, Image Analysis and Mass Spectrometry

The gels were stained by a modified CBB R250 staining protocol (Wang et al., 2007), and images were scanned with a Bio-Rad GS-800TM calibrated optical density scanner at 600 dpi. Images were then analyzed with Image Master 2D Platinum 5.0 (GE Healthcare) to integrate three replicates of the protein spots for each tapping. With the expression of the protein spot at the first tapping as a reference, the DE-protein spots (|ratio| ≥3.0, *T* value ≤.05)) were isolated from the SDS-PAGE gel, and sent to BPI Co., Ltd. (Beijing, China) for mass spectrometry identification on an ultraflex MALDI-TOF/TOF mass spectrometer instrument (Bruker Daltonics, Billerica, MA, United States).

### Bioinformatics Analysis

For DE-TDFs analysis, sequences of vectors and adaptors were first trimmed off by using the VecScreen program on the NCBI website (https://www.ncbi.nlm.nih.gov/tools/VecScreen). Then, the clean DE-TDF sequences were subjected for homology analysis to publicly available GenBank non-redundant sequences databases (http://www.ncbi.nlm.nih.gov) using the BLASTX program. Also, the Gene Ontology (http://amigo1.geneontology.org/cgi-bin/amigo/go.cgi) database was used to investigate the molecular function of each DE-TDF in the cell, which was used as the basis for functional classification.

### Statistical Analysis

At least three individual virgin or resting *Hevea* trees, with one tree serving as one biological replicate, were exploited. Data were analyzed by one-way ANOVA (SAS6.11) for comparison of TDF expressions across the five tappings. Student’s t-test was performed using the software embedded in Excel 2007 for comparison of TDF or protein spot abundance between the first tapping and any of the other four tappings. Differences were accepted as significant at *p* < .05 or .01.

## Data Availability

The original contributions presented in the study are publicly available in NCBI under accession numbers MZ935745-MZ936248.

## Abbreviations

2-DE, Two dimensional electrophoresis; AFLP, Amplified fragment length polymorphism; ATP, Adenosine triphosphate; CoA, Coenzyme A; DE-proteins, Differentially expressed proteins; DE-TDF, Differentially expressed transcript derived fragment; DNA, Deoxyribonucleic acid; dNTP, Deoxy-ribonucleoside triphosphate; EDTA, Ethylene diamine tetraacetic acid; IPG, Immobilized pH gradient; NADPH, Reduced form of nicotinamide-adenine dinucleotide phosphate; NIN, Alkaline/neutral invertase; NR, Natural rubber; PCR, Polymerase chain reaction; qRT-PCR, Quantitative reverse transcription PCR; RB, Rubber biosynthesis; REF, Rubber elongation factor; sqRT-PCR, Semi-quantitative reverse transcription PCR; SRPP, Small rubber particle protein; SUT, Sucrose transporter; TDF, Transcript derived fragment.
