# Supplementary material for: Transcript and Protein Profiling Provides Insights Into the Molecular Mechanisms of Harvesting-Induced Latex Production in Rubber Tree
Source: Front Genet. 2022 Feb 10;13:756270. doi: 10.3389/fgene.2022.756270 (PMC8869608; doi:10.3389/fgene.2022.756270)
Supplement: Supplementary file 1 [file Table4.DOC]

**Transcript and protein profiling provides insights into the molecular mechanisms of harvesting-induced latex production in rubber trees**

Yujie Fan^1, +^, Jiyan Qi^1, +^, Xiaohu Xiao^2, +^, Heping Li^1^, Jixian Lan^1^, Yacheng Huang^1^, Jianghua Yang^2^, Yi Zhang^1^, Shengmin Zhang^1^, Jun Tao^1^, Chaorong Tang^1,*^

^1^ Natural Rubber Cooperative Innovation Center of Hainan Province & Ministry of Education of PRC, Hainan University, Haikou 570228, China

^2^ Rubber Research Institute, Chinese Academy of Tropical Agricultural Sciences, Haikou 571101, China

^+^ These authors have contributed equally to this work.

^*^ Correspondence: [chaorongtang@126.com](mailto:chaorongtang@126.com); [chaorongtang@hainanu.edu.cn](mailto:chaorongtang@hainanu.edu.cn).

**Supplementary Table 4. Differentially expressed tapping-responsive protein spots identified by MALDI-TOF/TOF**

| **Functional group** | **Spot no. (pattern) ^a^** | **Protein name ^b^** | **GI no.** | **Exp. kDa/*p*I ^c^** | **Score ^d^** | **TOF/TOF sequence ^e^** |
| --- | --- | --- | --- | --- | --- | --- |
| Primary metabolism and energy | 356 (I) | glyceraldehyde-3- phosphate dehydrogenase | 262235239 | 38.4/6.77 | 127 | VPTVDVSVVDLTVR; LVTWYDNEWGYSTR |
|  | 372 (I) | glyceraldehyde 3-phosphate dehydrogenase | 255540341 | 40.0/7.88 | 115 |  |
|  | 593 (U) | phosphoglycerate mutase | 111162649 | 66.2/6.23 | 102 | ALEYENFDKFDR |
|  | 33 (U) | 2-phosphoglycerate dehydratase (enolase) | 14423688 | 37.0/5.65 | 200 | IEEELGSEAVYAGANFR |
|  | 229 (I) | 2-phosphoglycerate dehydratase (enolase) | 14423688 | 27.8/6.61 | 115 |  |
|  | 516 (I) | 2-phosphoglycerate dehydratase (enolase) | 14423688 | 53.2/5.03 | 265 | AAVPSGASTGIYEALELR |
|  | 534 (D) | 2-phosphoglycerate dehydratase (enolase) | 14423687 | 54.3/6.13 | 323 | VQIVGDDLLVTNPK; IEEELGAEAVYAGANFR; SGETEDTFIADLSVGLATGQIK |
|  | 539 (U) | 2-phosphoglycerate dehydratase (enolase) | 14423688 | 55.9/6.08 | 104 | NLVLPVPAFNVINGGSHAGNK |
|  | 591 (D) | 2-phosphoglycerate dehydratase (enolase) | 14423688 | 59.0/6.18 | 156 | AAVPSGASTGIYEALELR |
|  | 573 (U) | isoamylase | 31540845 | 62.3/5.71 | 80 | MRPNVAGLGRGR |
|  | 878 (I) | Phosphoenolpyruvate (PEP) carboxylase | 2626743 | 134.0/6.24 | 87 |  |
|  | 579 (U) | UDP-glucose pyrophosphorylase | 32527831 | 59.0/6.54 | 246 | VLQLETAAGAAIR;FFDHAIGINVPR |
|  | 813 (U) | UDP-glucose pyrophosphorylase | 32527831 | 106/6.05 | 77 | FFDHAIGINVPR |
|  | 350 (D) | Glucose and ribitol dehydrogenase | 110657778 | 38.0/5.35 | 180 | VPTVDVSVVDLTVR; LVTWYDNEWGYSTR |
|  | 603 (D) | UDP-glucose 6-dehydrogenase | 108708917 | 60.0/6.42 | 88 | LAANAFLAQR |
|  | 311 (D) | cysteine synthase | 255542380 | 37.0/6.42 | 234 | AFGAELVLTDPAR; TPLVYLNHVVDGCVAR |
|  | 791 (U) | 5-methyltetrahydropteroyltriglutamate-homocysteine methyltransferase | 255549601 | 96.0/6.96 | 149 | YGAGIGPGVYDIHSPR |
|  | 258 (U) | short chain dehydrogenase | 255552291 | 33.0/7.09 | 102 | AEGAKDPIAIPTDVGFEENCR |
| Secondary metabolism | 455 (U) | S-adenosylmethionine synthetase | 75299422 | 46.5/6.01 | 350 | TAAYGHFGR; FVIGGPHGDAGLTGR;  SIGFVSDDVGLDADKCK |
|  | 507 (D) | S-adenosylmethionine synthetase | 224130888 | 52.0/6.24 | 168 | FVIGGPHGDAGLTGR |
|  | 539 (U) | 1-aminocyclopropane-1-carboxylate synthase | 124020563 | 55.9/6.08 | 76 |  |
|  | 573 (U) | phytoene desaturase | 115392312 | 62.3/5.71 | 89 |  |
| Cell growth, division and structure | 375 (U) | caffeic acid methyltransferase | 110634239 | 39.9/5.84 | 203 |  |
|  | 376 (U) | caffeic acid methyltransferase | 110634239 | 39.9/6.02 | 74 |  |
|  | 808 (U) | Transitional endoplasmic reticulum ATPase | 255556938 | 115.8/6.13 | 208 | QSAPCVLFFDELDSIATQR;  LDQLIYIPLPDEDSR |
|  | 397 (D) | MYOSIN 1; motor/protein binding | 30685403 | 41.4/5.68 | 74 |  |
|  | 688 (I) | spindle disassembly related protein CDC48 | 98962497 | 67.3/6.23 | 95 |  |
|  | 690 (I) | spindle disassembly related protein CDC48 | [98962497](http://www.matrixscience.com/cgi/protein_view.pl?file=../data/20091127/FtmcCxSaR.dat&hit=10) | 104.2/5.50 | 178 | YTQGFSGADITEICQR; ELQETVQYPVEHPEKFEK |
|  | 537 (D) | actin | 32186912 | 55.0/5.68 | 307 | AVFPSIVGRPR; GEYDESGPSIVHR |
|  | 694 (I) | actin | 32186904 | 104.2/5.57 | 312 |  |
|  | 159 (D) | actin 1 (ACT1) | 149938964 | 24.0/6.61 | 92 |  |
|  | 593 (U) | alpha-tubulin 3 | 110638270 | 66.2/6.23 | 113 |  |
|  | 552 (I) | tubulin alpha chain | 255582570 | 57.0/5.57 | 259 | LISQIISSLTTSLR; AVFVDLEPTVIDEVR |
|  | 24 (I) | actin depolymerizing factor 4 | 224098343 | 18.4/5.87 | 74 | YAVYDFDYVTDENCQK |
| Stress and defense | 688 (I) | heat-shock protein | 1495251 | 67.3/6.23 | 72 |  |
|  | 778 (I) | heat shock protein | 33326375 | 94.0/5.55 | 111 | RAPFDLFDTR |
|  | 779 (U) | heat shock protein | 33326375 | 93.0/5.37 | 437 | LDAQPELFIR;  RAPFDLFDTR |
|  | 789 (U) | heat shock protein | 33326375 | 94.0/5.41 | 388 | LDAQPELFIR; ITLFLKEDQLEYLEER;  RAPFDLFDTR |
|  | 797 (D) | heat-shock protein | 33326375 | 98.0/5.59 | 310 | ADLVNNLGTIAR; ITLFLKEDQLEYLEER |
|  | 803 (I) | heat shock protein | 33326375 | 96.0/5.31 | 224 | RAPFDLFDTR;  ITLFLKEDQLEYLEER |
|  | 100 (D) | 17.1 kDa class II heat shock protein | 123550 | 16.6/4.19 | 84 | SGDIKVQVEDENVLLISGER |
|  | 750 (U) | heat-shock protein 70 | 186898205 | 85.0/5.67 | 116 | TTPSYVAFTDTER |
|  | 127 (I) | heat shock protein 90-2 | 208964722 | 19.0/5.36 | 226 | GIVDSEDLPLNISR; ITLFLKEDQLEYLEER |
|  | 618 (I) | similar to HSC70-1 | 225434984 | 73.7/6.58 | 486 | TTPSYVAFTDTER; ATAGDTHLGGEDFDNR;  NAVVTVPAYFNDSQR |
|  | 710 (I) | HSP91; ATP binding | 79321519 | 11.5/5.05 | 98 | ILTEFFGKEPR |
|  | 229 (I) | chitinase | 3451147 | 27.7/6.61 | 106 | VYLTAAPQCPFPDR |
|  | 631 (I) | latex cyanogenic beta glucosidase | 31580730 | 75.3/4.73 | 374 | NSLDFIGLNHYSSR; YFAIYADTCFASFGDR |
|  | 162 (I ) | IgE-binding protein MnSOD | 10862818 | 24.0/6.47 | 109 | LVVETTANQDPLVTK; NVRPDYLK |
|  | 331 (U) | allergenic isoflavone reductase-like protein | 110638104 | 37.0/5.65 | 126 |  |
|  | 173 (D) | latex protein allergen Hev b 7 | 6707018 | 24.6/5.57 | 279 | FIGANLTESK;  SLDCEDYYLR;  LVEIGTELLEKQESR |
|  | 396 (I) | latex protein allergen Hev b 7 | 6707018 | 42.0/5.36 | 83 |  |
|  | 436 (I) | latex allergen | 3288200 | 44.2/4.89 | 99 |  |
|  | 391(I) | desiccation-related protein At2g46140-like | 110656679 | 41.2/4.90 | 243 |  |
|  | 554 (I) | latex-abundant protein | 4235430 | 57.4/5.30 | 236 | SAEPGDLLFVHYSGHGTR |
|  | 628 (I) | latex-abundant protein | 4235430 | 63.0/5.41 | 368 | QTIQDAFESR; EFVDQVPHGCR;  SAEPGDLLFVHYSGHGTR |
|  | 85 (I) | prohevein | 2832430 | 15/8.00 | 95 | IVDQCSNGGLDLDVNVFR |
|  | 182 (D) | cysteine proteinase inhibitor | 110667114 | 25.0/7.53 | 213 |  |
| Transcription, translation and protein fate | 247 (D) | RNA polymerase beta subunit-2 | 222139885 | 18.4/5.87 | 73 |  |
|  | 315 (I) | elongation factor 1-gamma 2 | 226530767 | 36.1/6.30 | 96 | SFTSEFPHVER |
|  | 534 (D) | elongation factor 1 gamma-like protein | 29367381 | 54.3/6.12 | 132 | SFTSEFPHVER |
|  | 375 (U) | O-methyltransferase | 15218133 | 39.9/5.84 | 84 | NPEAPVLLDR |
|  | 376 (U) | O-methyltransferase | 30687192 | 39.9/6.02 | 72 | NPEAPVLLDR |
|  | 645 (U) | acyl-peptide hydrolase-like | 9759033 | 78.0/5.14 | 130 | VLVSGNDFYAFPR |
|  | 652 (U) | glycyl-tRNA synthetase | 255543218 | 82.0/6.13 | 111 | VFTPSVIEPSFGIGR |
|  | 70 (U) | s24-like peptidase | 255070443 | 14.0/6.51 | 72 |  |
|  | 476 (D) | 3'-5' exonuclease | 255550866 | 49/5.86 | 130 | YCGISYLEKEEVR |
| Signal transduction | 33 (U) | serine/threonine-protein kinase C41C4.4 precursor | 116000725 | 61.5/4.81 | 74 | SALTEAVIGAER |
|  | 805 (U) | Phospholipase D alpha 1 | 255553165 | 100.0/5.94 | 251 | SIQDAYIHAIRR;  SIDGGAAFGFPETPEDAAR |
|  | 809 (U) | Phopholipase D alpha 1 | 255553165 | 106.0/6.15 | 163 | SIDGGAAFGFPETPEDAAR |
|  | 811 (U) | Phospholipase D alpha 1 | 255553165 | 106.0/6.26 | 199 | SIDGGAAFGFPETPEDAAR |
| Rubber biosynthesis | 57 (I) | Rubber elongation factor protein | 132270 | 10.9/4.38 | 96 |  |
|  | 149 (U) | Small rubber particle protein | 14423933 | 21.7/4.08 | 76 |  |
|  | 163 (U) | Small rubber particle protein | 14423933 | 24.0/5.22 | 322 | AEQYAVITWR; QVSAQTYSVAQDAPR |
|  | 723 (D) | Small rubber particle protein | 14423933 | 79.0/6.91 | 176 | AEQYAVITWR; AAGVYAVDSFSTLYLYAK |
|  | 730 (I) | Small rubber particle protein | 14423933 | 81.0/5.04 | 306 | AEQYAVITWR; QVSAQTYSVAQDAPR |
|  | 858 (U) | Small rubber particle protein | 14423933 | 121.0/5.70 | 116 |  |
|  | 442 (I) | acetyl-CoA C-acetyltransferase | 164604962 | 44.0/7.58 | 151 | GIAAQESGAFAWEIVPVEVSGGR |
|  | 461 (D) | acetyl-CoA C-acetyltransferase | 164604962 | 45.0/6.92 | 168 | ILVTLLGVLR |
| Unknown proteins | 21 (I) | hypothetical protein CHLREDRAFT_205506 | 159472458 | 38.2/6.01 | 83 |  |
|  | 653 (U) | hypothetical protein OsI_34730 | 218185055 | 81.5/6.17 | 101 |  |
|  | 364 (I) | Os12g0226700 | 115487890 | 40.0/5.87 | 78 |  |
|  | 25 (U) | predicted protein | 255075425 | 61.4/4.81 | 71 |  |
|  | 247 (D) | predicted protein | 224126739 | 30.3/5.74 | 74 |  |
|  | 371 (I) | predicted protein | 168049341 | 39.6/4.50 | 72 |  |
|  | 556 (U) | predicted protein | 224132896 | 57.6/6.35 | 72 |  |
|  | 559 (U) | predicted protein | 145348964 | 66.5/5.09 | 89 |  |
|  | 615 (I) | predicted protein | 224135733 | 72.0/6.24 | 73 | LLVHESIASEFLDR |
|  | 639 (D) | hypothetical protein | 147770841 | 65.3/4.70 | 76 |  |
|  | 858 (I) | hypothetical protein | 225442661 | 121/5.70 | 72 |  |

^a^ The expression patterns of each protein spot on 2-DE gels along the five tappings were classified into three types: up-regulated (U), down-regulated (D), and irregularly-regulated (I).

^b^ The name of proteins was identified by MALDI-TOF MS.

^c^ Experimental mass (kDa) and *p*I of the protein spots were determined based on their 2-DE appearance.

^d^ The scores greater than 71 were considered positive when searching NCBI nr protein databases.

^e^ The peptide sequences were obtained by MALDI-TOF/TOF tandem MS with 2-4 peptides selected from positive MALDI-TOF MS.
