# Supplementary material for: Transcript and Protein Profiling Provides Insights Into the Molecular Mechanisms of Harvesting-Induced Latex Production in Rubber Tree
Source: Front Genet. 2022 Feb 10;13:756270. doi: 10.3389/fgene.2022.756270 (PMC8869608; doi:10.3389/fgene.2022.756270)
Supplement: Supplementary file 2 [file Table6.doc]

**Transcript and protein profiling provides insights into the molecular mechanisms of harvesting-induced latex production in rubber tree**

Yujie Fan^1, +^, Jiyan Qi^1, +^, Xiaohu Xiao^2, +^, Heping Li^1^, Jixian Lan^1^, Yacheng Huang^1^, Jianghua Yang^2^, Yi Zhang^1^, Shengmin Zhang^1^, Jun Tao^1^, Chaorong Tang^1,*^

^1^ Natural Rubber Cooperative Innovation Center of Hainan Province & Ministry of Education of PRC, Hainan University, Haikou 570228, China

^2^ Rubber Research Institute, Chinese Academy of Tropical Agricultural Sciences, Haikou 571101, China

^+^ These authors have contributed equally to this work.

^*^ Correspondence: [chaorongtang@126.com](mailto:chaorongtang@126.com); [chaorongtang@hainanu.edu.cn](mailto:chaorongtang@hainanu.edu.cn).

**Supplementary Table 6. The overlap between the DE-TDFs and DE-protein spots**

| **No.^a)^** | **Function annotation** | **DE-TDFs** | **DE-protein spots** |
| --- | --- | --- | --- |
| 1 | small rubber particle protein | M1-A10-2, M12-A6-4 | 163(U), 723(D), 730(Ir) |
| 2 | tubulin alpha-3 chain-like protein | M6-A10-4 | 552(Ir) |
| 3 | latex abundant family protein | M6-A7-1, M8-A7-2 | 554(Ir), 628(Ir) |
| 4 | beta-glucosidase | M12-A10-4 | 631(Ir) |
| 5 | cell division protein | M8-A8-1 | 690(Ir), 808(U) |
| 6 | cysteine synthase like protein | M12-A8-3 | 311(D) |
| 7 | elongation factor | M7-A10-3, M10-A6-1 | 315(Ir), 534(D) |
| 8 | heat shock protein | M10-A9-2, M16-A6-1 | 127(Ir), 618(Ir), 710(Ir), 750(U), 778(Ir),  779(U), 789(U), 797(D), 803(Ir) |
| 9 | phospholipase | M15-A6-4 | 805(U), 809(U), 811(U) |
| 10 | wound-induced protein | M15-A11-1 | 85(Ir) |

a): 1-3 represent the DE-TDFs and DE-protein spots belonging to the same gene; 4-10 represent the DE-TDFs and DE-protein spots belonging to the same gene family but distinct genes.
