# Supplementary material for: Transcript and Protein Profiling Provides Insights Into the Molecular Mechanisms of Harvesting-Induced Latex Production in Rubber Tree
Source: Front Genet. 2022 Feb 10;13:756270. doi: 10.3389/fgene.2022.756270 (PMC8869608; doi:10.3389/fgene.2022.756270)
Supplement: Supplementary file 3 [file Table3.DOC]

**Transcript and protein profiling provides insights into the molecular mechanisms of harvesting-induced latex production in rubber trees**

Yujie Fan^1, +^, Jiyan Qi^1, +^, Xiaohu Xiao^2, +^, Heping Li^1^, Jixian Lan^1^, Yacheng Huang^1^, Jianghua Yang^2^, Yi Zhang^1^, Shengmin Zhang^1^, Jun Tao^1^, Chaorong Tang^1,*^

^1^ Natural Rubber Cooperative Innovation Center of Hainan Province & Ministry of Education of PRC, Hainan University, Haikou 570228, China

^2^ Rubber Research Institute, Chinese Academy of Tropical Agricultural Sciences, Haikou 571101, China

^+^ These authors have contributed equally to this work.

^*^ Correspondence: [chaorongtang@126.com](mailto:chaorongtang@126.com); [chaorongtang@hainanu.edu.cn](mailto:chaorongtang@hainanu.edu.cn).

**Supplementary Table 3. Functional annotation and cDNA-AFLP profiles of the irregularly-regulated DE-TDFs**

| **DE-TDFs ^a)^** | **Size**  **(bp)** | **Function annotation ^b)^[species]** | **Accession number** | **E-Value** | **cDNA-AFLP gel picture ^c)^** |
| --- | --- | --- | --- | --- | --- |
| **Primary metabolism (3)** | | | | | |
| M8-A9-3 | 287 | GDP-L-galactose phosphorylase [*Malpighia* *glabra*] | ACG75920 | 4E-25 | 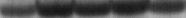 |
| M4-A6-3 | 250 | omega-6 fatty acid desaturase [*Hevea* *brasiliensis*] | AAY87459 | 2E-20 | 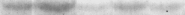 |
| M3-A10-1 | 539 | beta-ketoacyl-coa synthase family protein  [*Populus* *trichocarpa*] | XP_002308642 | 1E-91 | 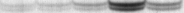 |
| **Energy (7)** | | | | | |
| M2-A5-5 | 209 | adenylate kinase 1 chloroplast [*Ricinus* *communis*] | XP_002511210 | 3E-11 | 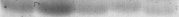 |
| M3-A8-4 | 299 | pyruvate dehydrogenase, putative [*Ricinus* *communis*] | XP_002512633 | 1E-38 | 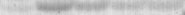 |
| M4-A5-5 | 350 | NADP-dependent glyceraldehyde-3-phosphate  dehydrogenase, putative [*Ricinus* *communis*] | XP_002523495 | 2E-26 | 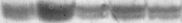 |
| M5-A5-1 | 451 | G6PD1 [*Actinidia* *chinensis*] | ABR45722 | 1E-79 | 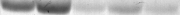 |
| M11-A7-5 | 166 | Iron-sulfur assembly protein IscA, chloroplast precursor, putative [*Ricinus* *communis*] | XP_002532181 | 4E-44 | 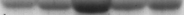 |
| M12-A6-6 | 80 | cytochrome P450, putative [*Ricinus* *communis*] | XP_002529227 | 1E-130 | 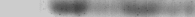 |
| M12-A7-2 | 271 | phosphofructokinase, putative [*Ricinus* *communis*] | XP_002525025 | 1E-140 | 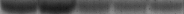 |
| **Cell growth and division (3)** | | | | | |
| M9-A11-2 | 376 | SAUR family protein [*Populus* *trichocarpa*] | XP_002318465 | 4E-34 | 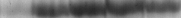 |
| M9-A10-2 | 363 | SAUR family protein [*Populus* *trichocarpa*] | XP_002303770 | 2E-06 | 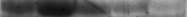 |
| M14-A7-2 | 274 | B2 protein, putative [*Ricinus* *communis*] | XP_002509516 | 7E-27 | 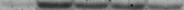 |
| **Transcription and protein synthesis (20)** | | | | | |
| M5-A6-1 | 528 | Transcriptional corepressor SEUSS [*Ricinus* *communis*] | XP_002520244 | 7E-62 | 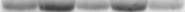 |
| M11-A5-3 | 175 | RNA processing factor 1-like [*Solanum* *tuberosum*] | ABB86266 | 5E-13 | 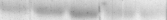 |
| M11-A5-7 | 84 | OB-fold nucleic acid binding domain containing protein [*Zea* *mays*] | NP_001146919 | 1E-17 | 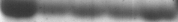 |
| M14-A6-1 | 371 | histone deacetylase hda1, putative [*Ricinus* *communis*] | XP_002514660 | 4E-31 | 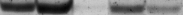 |
| M3-A10-4 | 315 | pentatricopeptide repeat-containing protein, putative  [*Ricinus* *communis*] | XP_002522167 | 3E-26 | 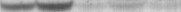 |
| M10-A5-1 | 180 | pentatricopeptide repeat-containing protein, putative  [*Ricinus* *communis*] | XP_002510403 | 3E-09 | 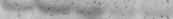 |
| M5-A5-3 | 360 | transcription regulator, putative [*Ricinus* *communis*] | XP_002518574 | 8E-08 | 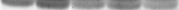 |
| M5-A10-4 | 278 | zinc finger protein, putative [*Ricinus* *communis*] | XP_002525195 | 1E-09 | 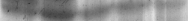 |
| M3-A8-1 | 358 | zinc finger (C3HC4-type RING finger) family protein  [*Arabidopsis* *thaliana*] | NP_566208 | 1E-35 | 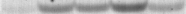 |
| M2-A5-6 | 145 | RNA-binding region-containing protein[*Ricinus* *communis*] | XP_002523906 | 1E-15 | 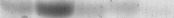 |
| M1-A10-1 | 288 | RNA recognition motif (RRM)-containing protein  [*Arabidopsis* *thaliana*] | NP_190296 | 6E-75 | 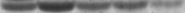 |
| M5-A10-1 | 442 | nucleic acid binding protein [*Ricinus* *communis*] | XP_002527351 | 9E-41 | 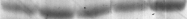 |
| M4-A5-7 | 280 | DNA binding protein, putative [*Ricinus* *communis*] | XP_002518757 | 1E-53 | 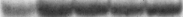 |
| M3-A6-9 | 99 | Heterogeneous nuclear ribonucleoprotein A1, putative  [*Ricinus* *communis*] | XP_002521392 | 1E-63 | 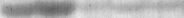 |
| M3-A7-9 | 160 | step II splicing factor slu7 [*Ricinus* *communis*] | XP_002524613 | 0 | 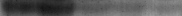 |
| M16-A11-3 | 222 | NAC domain-containing protein 21/22[*Ricinus* *communis*] | XP_002514736 | 2E-16 | 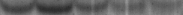 |
| M7-A8-1 | 310 | 50S ribosomal protein L19 [*Ricinus* *communis*] | XP_002531788 | 2E-18 | 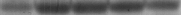 |
| M8-A10-1 | 722 | nucleolar protein nop56, putative [*Ricinus* *communis*] | XP_002511213 | 5E-93 | 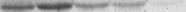 |
| M16-A8-1 | 416 | polyadenylate-binding protein [*Ricinus* *communis*] | XP_002518733 | 2E-49 | 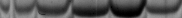 |
| M11-A7-3 | 212 | alanyl-tRNA synthetase [*Acinetobacter* *sp*. RUH2624] | ZP_05824178 | 5E-21 | 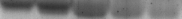 |
| **Protein degradation and storage (7)** | | | | | |
| M4-A6-1 | 656 | ATP-dependent protease Clp ATPase subunit[*Zea* *mays*] | NP_001147667 | 5E-50 | 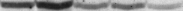 |
| M6-A7-2 | 493 | 20S proteasome alpha subunit E [*Glycine* *max*] | Q9M4T8 | 1E-70 | 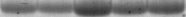 |
| M11-A7-8 | 106 | ubiquitin-conjugating enzyme m [*Ricinus* *communis*] | XP_002525098 | 1E-12 | 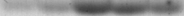 |
| M11-A11-1 | 337 | Vacuolar-processing enzyme precursor  [*Ricinus* *communis*] | XP_002516472 | 1E-43 | 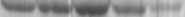 |
| M11-A7-2 | 326 | plant ubiquilin, putative [*Ricinus* *communis*] | XP_002521706 | 6E-22 | 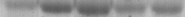 |
| M8-A7-1 | 697 | sodium symporter-related [*Arabidopsis* *thaliana*] | NP_001117264 | 1E-49 | 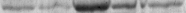 |
| M2-A11-2 | 316 | Ubiquitin-60S ribosomal protein L40  [*Nicotiana* *sylvestris*] | P49636 | 1E-47 | 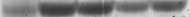 |
| **Transporters and intracellular transport (7)** | | | | | |
| M5-A7-1 | 407 | possible peptide-transporting ATPase  [*Sphingobacterium* *spiritivorum* ATCC 33300] | ZP_03969151 | 1E-32 | 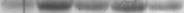 |
| M3-A12-1 | 666 | RAB6A [*Arabidopsis* *thaliana*] | NP_181989 | 6E-59 | 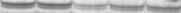 |
| M7-A5-1* |  | Early nodulin 55-2 precursor [*Ricinus* *communis*] | XP_002522863 | 4E-48 | 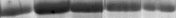 |
| M2-A6-2 | 346 | AGD13 (ARF-GAP domain 13) [*Arabidopsis* *thaliana*] | NP_567292 | 4E-15 | 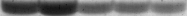 |
| M10-A8-4 | 99 | Vesicle-associated membrane protein[*Ricinus* *communis*] | XP_002521112 | 1E-08 | 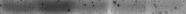 |
| M4-A5-6 | 317 | coatomer delta subunit, putative [Ricinus communis] | XP_002526581 | 2E-33 | 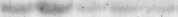 |
| M11-A11-5 | 106 | vacuolar ATP synthase proteolipid subunit 1, 2, 3, putative [*Ricinus* *communis*] | XP_002513751 | 5E-56 | 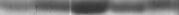 |
| **Signal transduction (12)** | | | | | |
| M2-A5-4 | 241 | nucleoredoxin, putative [*Ricinus* *communis*] | XP_002525369 | 5E-17 | 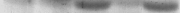 |
| M2-A8-1 | 418 | kinase, putative [*Ricinus* *communis*] | XP_002533426 | 2E-41 | 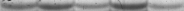 |
| M2-A8-3 | 267 | putative 7-transmembrane G-protein-coupled kinase  [*Solanum* *chacoense*] | AAF75794 | 2E-97 | 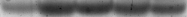 |
| M7-A6-2 | 453 | Sphingosine kinase, putative [*Ricinus* *communis*] | XP_002524674 | 9E-43 | 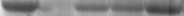 |
| M7-A10-1 | 707 | serine-threonine protein kinase, plant-type, putative  [*Ricinus* *communis*] | XP_002524773 | 1E-97 | 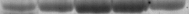 |
| M10-A7-1 | 349 | xenotropic and polytropic murine leukemia virus receptor ids-4, putative [*Ricinus* *communis*] | XP_002524498 | 2E-31 | 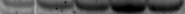 |
| M10-A8-2 | 303 | Phospholipase C 3 precursor [*Ricinus* *communis*] | XP_002524007 | 8E-08 | 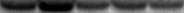 |
| M11-A8-5 | 208 | ASK-dzeta (ASK4) [*Vitis* *vinifera*] | XP_002281788 | 3E-32 | 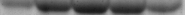 |
| M6-A10-2 | 360 | serine/threonine protein phosphatase [*Ricinus* *communis*] | XP_002521968 | 1E-54 | 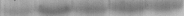 |
| M8-A5-1 | 435 | WD-repeat protein, putative [*Ricinus* *communis*] | XP_002511220 | 2E-40 | 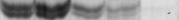 |
| M10-A6-2 | 270 | leucine aminopeptidase, putative [*Ricinus* *communis*] | XP_002529380 | 2E-34 | 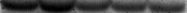 |
| M11-A11-3 | 211 | serine-threonine protein kinase, plant-type, putative  [*Ricinus* *communis*] | XP_002530200 | 2E-14 | 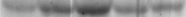 |
| **Stress and defense (7)** | | | | | |
| M3-A12-2 | 408 | Glucan endo-1,3-beta-glucosidase precursor, putative  [*Ricinus* *communis*] | XP_002518468 | 1E-53 | 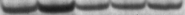 |
| M11-A12-4 | 112 | Arylacetamide deacetylase[*Ricinus* *communis*] | XP_002523490 | 2E-10 | 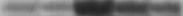 |
| M14-A10-2 | 574 | glutamate dehydrogenase, putative [*Ricinus* *communis*] | XP_002515882 | 7E-86 | 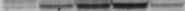 |
| M9-A7-2 | 245 | MlrC family protein [*Acinetobacter* *lwoffii* SH145] | ZP_06068991 | 2E-22 | 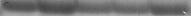 |
| M4-A5-4 | 434 | aig1, putative [*Ricinus* *communis*] | XP_002515131 | 5E-52 | 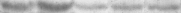 |
| M10-A6-3 | 159 | Immunoglobulin/major histocompatibility complex;  Tetratricopeptide-like helical [*Medicago* *truncatula*] | ABP03850 | 4E-13 | 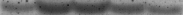 |
| M6-A12-3 | 220 | (S)-N-methylcoclaurine 3'-hydroxylase  [*Eschscholzia* *californica*] | AAC39454 | 6E-13 | 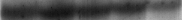 |
| **Secondary metabolism (1)** | | | | | |
| M9-A9-3 | 255 | farnesyl-diphosphate farnesyltransferase  [*Ricinus* *communis*] | XP_002512982 | 3E-25 | 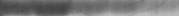 |
| **Rubber biosynthesis (1)** | | | | | |
| M8-A5-6 | 187 | inorganic pyrophosphatase [*Ricinus* *communis*] | XP_002531333 | 2E-117 | 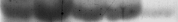 |
| **Unclassified proteins (7)** | | | | | |
| M11-A7-7 | 127 | ATP binding protein, putative [*Ricinus* *communis*] | XP_002524736 | 0 | 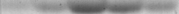 |
| M11-A11-2 | 296 | ATP binding protein, putative [*Ricinus* *communis*] | XP_002524736 | 2E-24 | 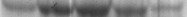 |
| M11-A12-2 | 125 | ATP binding protein, putative [*Ricinus communis*] | XP_002526190 | 3E-84 | 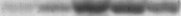 |
| M14-A12-3 | 155 | leucine-rich repeat family protein-like protein  [*Helianthus annuus*] | ACO56656 | 7E-06 | 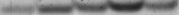 |
| M11-A9-2 | 353 | developmentally regulated GTP-binding protein, putative  [*Ricinus communis*] | XP_002532275 | 5E-28 | 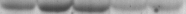 |
| M2-A10-5 | 184 | catalytic, putative [*Ricinus communis*] | XP_002528256 | 2E-09 | 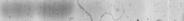 |
| M6-A12-5 | 154 | catalytic, putative [*Ricinus communis*] | XP_002524233 | 3E-87 | 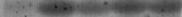 |
| **Predicted proteins (15)** | | | | | |
| M1-A5-2 | 280 | conserved hypothetical protein [*Ricinus communis*] | XP_002521048 | 6E-13 | 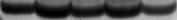 |
| M4-A10-1 | 345 | conserved hypothetical protein [*Ricinus communis*] | XP_002523743 | 3E-24 | 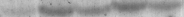 |
| M10-A10-3 | 316 | conserved hypothetical protein [*Ricinus communis*] | XP_002515458 | 4E-21 | 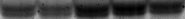 |
| M11-A8-6 | 141 | predicted protein [*Populus trichocarpa*] | XP_002337272 | 1E-12 | 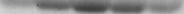 |
| M11-A11-4 | 114 | conserved hypothetical protein [*Ricinus communis*] | XP_002531967 | 8E-70 | 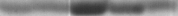 |
| M7-A9-3 | 293 | conserved hypothetical protein [*Ricinus communis*] | XP_002527937 | 2E-37 | 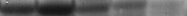 |
| M14-A8-2 | 369 | predicted protein [*Populus trichocarpa*] | XP_002325938 | 2E-18 | 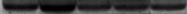 |
| M5-A5-2 | 359 | conserved hypothetical protein [*Ricinus communis*] | XP_002524975 | 6E-20 | 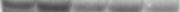 |
| M13-A9-4 | 134 | predicted protein [*Populus trichocarpa*] | XP_002300393 | 7E-11 | 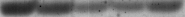 |
| M11-A9-5 | 150 | PREDICTED: hypothetical protein [*Vitis vinifera*] | XP_002263192 | 8E-56 | 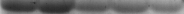 |
| M11-A8-4 | 248 | hypothetical protein [*Vitis vinifera*] | CAN62161 | 1E-56 | 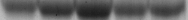 |
| M15-A6-3 | 245 | PREDICTED: hypothetical protein [*Vitis vinifera*] | XP_002265330 | 1E-156 | 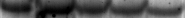 |
| M13-A9-3 | 202 | PREDICTED: hypothetical protein [*Vitis vinifera*] | XP_002279853 | 5E-06 | 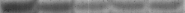 |
| M11-A12-3 | 149 | conserved hypothetical protein [*Ricinus communis*] | XP_002518932 | 7E-11 | 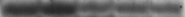 |
| M6-A8-5 | 140 | conserved hypothetical protein [*Ricinus communis*] | XP_002515303 | 2E-108 | 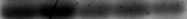 |
| **No hit sequence (18)** | | | | | |
| M6-A7-4 | 189 |  |  |  | 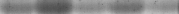 |
| M11-A7-10 | 89 |  |  |  | 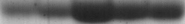 |
| M11-A9-3 | 227 |  |  |  | 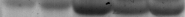 |
| M12-A8-3 | 235 |  |  |  | 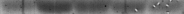 |
| M12-A8-4 | 153 |  |  |  | 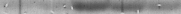 |
| M11-A7-6 | 144 |  |  |  | 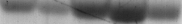 |
| M2-A7-4 | 222 |  |  |  | 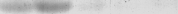 |
| M3-A10-11 | 201 |  |  |  | 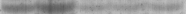 |
| M4-A10-3 | 129 |  |  |  | 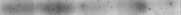 |
| M8-A11-2 | 194 |  |  |  | 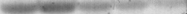 |
| M11-A5-5 | 98 |  |  |  |  |
| M11-A5-6 | 98 |  |  |  |  |
| M10-A5-3 | 162 |  |  |  |  |
| M3-A8-9 | 128 |  |  |  |  |
| M3-A11-5 | 344 |  |  |  |  |
| M7-A7-5 | 147 |  |  |  |  |
| M7-A8-4 | 165 |  |  |  |  |
| M2-A5-3 | 237 |  |  |  |  |

a): DE-TDFs number, including primer combinations for selective amplification. M: restriction enzyme *Mse* I, A: restriction enzyme *Apo* I. For example: M1-A5-1, using *Mse* I-SP_1_ and *Apo* I-SP_5_ selective primers for screening, obtaining the first DE-TDF.

b): DE-TDFs function annotation results in NCBI (<http://blast.ncbi.nlm.nih.gov/Blast.cgi>), “[ ]” is the corresponding species.

c): The expression pattern of DE-TDFs analyzed by cDNA-AFLP, showing the expression level in the first five tapping from left to right.
