# Supplementary material for: Transcript and Protein Profiling Provides Insights Into the Molecular Mechanisms of Harvesting-Induced Latex Production in Rubber Tree
Source: Front Genet. 2022 Feb 10;13:756270. doi: 10.3389/fgene.2022.756270 (PMC8869608; doi:10.3389/fgene.2022.756270)
Supplement: Supplementary file 4 [file Table1.DOC]

**Transcript and protein profiling provides insights into the molecular mechanisms of harvesting-induced latex production in rubber trees**

Yujie Fan^1, +^, Jiyan Qi^1, +^, Xiaohu Xiao^2, +^, Heping Li^1^, Jixian Lan^1^, Yacheng Huang^1^, Jianghua Yang^2^, Yi Zhang^1^, Shengmin Zhang^1^, Jun Tao^1^, Chaorong Tang^1,*^

^1^ Natural Rubber Cooperative Innovation Center of Hainan Province & Ministry of Education of PRC, Hainan University, Haikou 570228, China

^2^ Rubber Research Institute, Chinese Academy of Tropical Agricultural Sciences, Haikou 571101, China

^+^ These authors have contributed equally to this work.

^*^ Correspondence: [chaorongtang@126.com](mailto:chaorongtang@126.com); [chaorongtang@hainanu.edu.cn](mailto:chaorongtang@hainanu.edu.cn).

**Supplementary Table 1. Functional annotation and cDNA-AFLP profiles of the up-regulated DE-TDFs**

| **DE-TDFs ^a)^** | **Size (bp)** | **Function annotation ^b)^[species]** | **Accession number** | **E-Value** | **cDNA-AFLP gel picture ^c)^** |
| --- | --- | --- | --- | --- | --- |
| **Primary metabolism (15)** | | | | | |
| M8-A8-3 | 108 | Adenine phosphoribosyltransferase  [*Ricinus* *communis*] | XP_002510107 | 1E-87 | 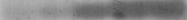 |
| M1-A8-1 | 237 | triacylglycerol lipase, putative [*Ricinus* *communis*] | XP_002533321 | 3E-23 | 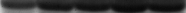 |
| M7-A9-5 | 155 | dolichyldiphosphatase, putative [*Ricinus* *communis*] | XP_002525172 | 3E-09 | 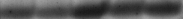 |
| M7-A10-5 | 232 | arginine decarboxylase, putative [*Ricinus* *communis*] | XP_002513004 | 4E-15 | 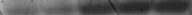 |
| M13-A11-5 | 393 | pyruvate kinase, putative [*Ricinus* *communis*] | XP_002523396 | 1E-44 | 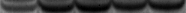 |
| M15-A7-2 | 166 | acyl carrier protein, putative [*Ricinus* *communis*] | XP_002516860 | 3E-09 | 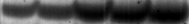 |
| M5-A12-5 | 242 | acyl carrier protein [*Ricinus* *communis*] | XP_002512285 | 2E-39 | 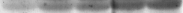 |
| M15-A5-1 | 264 | phosphatidic acid phosphatase-related  [*Arabidopsis* *thaliana*] | NP_201446 | 5E-21 | 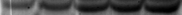 |
| M3-A6-8 | 222 | SAT5 [*Zea* *mays*] | ACG37675 | 6E-26 | 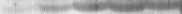 |
| M7-A6-1 | 528 | cdp-diacylglycerol--glycerol-3-phosphate  [*Ricinus* *communis*] | XP_002518507 | 6E-26 | 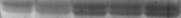 |
| M14-A7-1 | 321 | VTC2-like protein [*Actinidia* *chinensis*] | ABP65665 | 3E-38 | 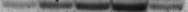 |
| M4-A8-4 | 129 | VTC2, putative, expressed [*Oryza* *sativa*] | ABA99379 | 3E-53 | 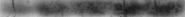 |
| M16-A7-4 | 125 | CNX7; catalytic [*Arabidopsis* *thaliana*] | NP_567352 | 7E-25 | 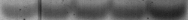 |
| M15-A12-1 | 226 | Very-long-chain 3-ketoacyl-CoA synthase  [*Medicago* *truncatula*] | ABD32702 | 1E-30 | 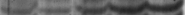 |
| M16-A7-1 | 646 | neutral/alkaline invertase [*Manihot* *esculenta*] | ABA08442 | 6E-114 | 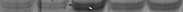 |
| **Energy (8)** | | | | | |
| M15-A7-3 | 146 | 2-oxo acid dehydrogenase, lipoyl-binding site  [*Medicago* *truncatula*] | ABC75361 | 7E-37 | 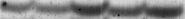 |
| M14-A7-4 | 145 | fructokinase, putative [*Ricinus* *communis*] | XP_002533363 | 1E-07 | 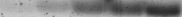 |
| M14-A5-2 | 383 | ferrochelatase, putative [*Ricinus* *communis*] | XP_002513282 | 5E-52 | 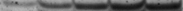 |
| M10-A12-4 | 156 | ferrochelatase, putative [*Ricinus* *communis*] | XP_002513282 | 3E-95 | 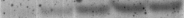 |
| M10-A12-1 | 412 | cytochrome B5 isoform 1, putative  [*Ricinus* *communis*] | XP_002518220 | 2E-33 | 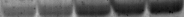 |
| M13-A8-2 | 525 | pyruvate dehydrogenase, putative  [*Ricinus* *communis*] | XP_002520198 | 2E-27 | 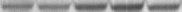 |
| M15-A8-3 | 216 | glyceraldehyde-3-phosphate dehydrogenase  [*Nicotiana* *tabacum*] | CAB39974 | 1E-109 | 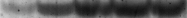 |
| M7-A8-2 | 229 | iron-sulfur cluster assembly protein  [*Ricinus* *communis*] | XP_002528740 | 3E-63 | 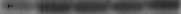 |
| **Cell growth and division (11)** | | | | | |
| M15-A11-3 | 151 | nucleolysin tia-1, putative [*Ricinus* *communis*] | XP_002529199 | 1E-06 | 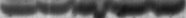 |
| M4-A8-2 | 323 | Alpha-expansin 20 precursor [*Ricinus* *communis*] | XP_002514405 | 6E-18 | 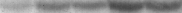 |
| M15-A8-1 | 445 | histone H3.2 [*Arabidopsis* *thaliana*] | NP_001078516 | 1E-49 | 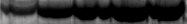 |
| M16-A9-3 | 297 | histone H2B, putative [*Arabidopsis* *thaliana*] | NP_180440 | 8E-43 | 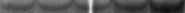 |
| M12-A5-2 | 260 | histone h1/h5, putative [*Ricinus* *communis*] | XP_002520997 | 2E-10 | 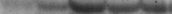 |
| M15-A10-3 | 368 | histone h2a, putative [*Ricinus* *communis*] | XP_002520711 | 1E-33 | 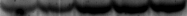 |
| M15-A9-3 | 126 | Single-strand binding protein family [*Oryza* *sativa*] | ABF97593 | 9E-07 | 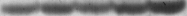 |
| M8-A8-1 | 572 | Cell division protein ftsZ [*Ricinus* *communis*] | XP_002531210 | 1E-67 | 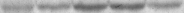 |
| M2-A5-7 | 197 | DNA polymerase I, putative [*Ricinus* *communis*] | XP_002530572 | 2E-88 | 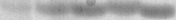 |
| M7-A11-3 | 89 | H3/H4 histone acetyltransferase  [*Arabidopsis* *thaliana*] | NP_565157 | 2E-38 | 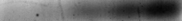 |
| M1-A7-3 | 497 | MFP1 attachment factor 1 [*Glycine* *max*] | AAF63659 | 1E-32 | 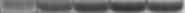 |
| **Transcription and protein synthesis (41)** | | | | | |
| M3-A10-7 | 250 | pentatricopeptide repeat-containing protein, putative [*Ricinus* *communis*] | XP_002523226 | 2E-19 | 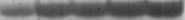 |
| M2-A8-2 | 344 | pentatricopeptide repeat-containing protein, putative  [*Ricinus* *communis*] | XP_002528283 | 1E-15 | 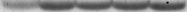 |
| M2-A9-1 | 402 | DNA binding/zinc ion binding protein  [*Gossypium* *hirsutum*] | ABD65463 | 2E-25 | 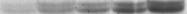 |
| M6-A11-4 | 366 | DNA binding protein, putative [*Ricinus* *communis*] | XP_002529155 | 1E-31 | 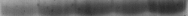 |
| M3-A10-6 | 292 | DNA binding protein, putative [*Ricinus* *communis*] | XP_002514030 | 3E-59 | 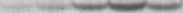 |
| M2-A12-3 | 220 | zinc finger protein, putative [*Ricinus* *communis*] | XP_002526088 | 4E-90 | 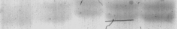 |
| M13-A11-2 | 504 | zinc finger (C2H2 type) family protein  [*Arabidopsis* *thaliana*] | NP_187658 | 7E-25 | 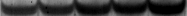 |
| M12-A9-1 | 501 | AN1-like transcription factor [*Glycine* *max*] | ADK25058 | 3E-42 | 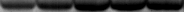 |
| M3-A6-5 | 285 | zinc finger (Ran-binding) family protein  [*Arabidopsis* *thaliana*] | NP_175290 | 2E-16 | 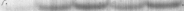 |
| M13-A6-5 | 106 | phd/F-box containing protein [*Ricinus* *communis*] | XP_002528085 | 1E-12 | 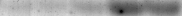 |
| M6-A12-1 | 759 | DNA-directed RNA polymerase II subunit, putative  [*Ricinus* *communis*] | XP_002518700 | 8E-56 | 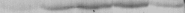 |
| M11-A6-4 | 114 | DNA-directed RNA polymerase III  [*Ricinus* *communis*] | XP_002523512 | 8E-13 | 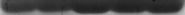 |
| M12-A6-3 | 210 | DNA-directed RNA polymerase, subunit C11/M/9  [*Medicago* *truncatula*] | ABD32320 | 1E-22 | 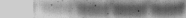 |
| M7-A7-4 | 161 | small nuclear ribonucleoprotein f [*Ricinus* *communis*] | XP_002530746 | 1E-23 | 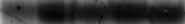 |
| M11-A12-1 | 176 | Ocs element-binding factor [*Ricinus* *communis*] | XP_002515285 | 5E-12 | 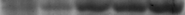 |
| M11-A7-4 | 176 | Ocs element-binding factor [*Ricinus* *communis*] | XP_002515285 | 2E-15 | 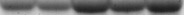 |
| M12-A7-5 | 105 | GAGA-binding transcriptional activator BBR/BPC1-like[*Vitis* *vinifera*] | ACC64525 | 5E-10 | 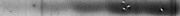 |
| M12-A8-5 | 126 | Mago Nashi-like protein [*Euphorbia* *lagascae*] | CAA70006 | 7E-52 | 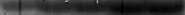 |
| M12-A10-5 | 199 | MYC1 [*Hevea* *brasiliensis*] | ACF05947 | 5E-26 | 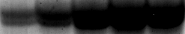 |
| M12-A12-2 | 91 | transcription factor, putative [*Ricinus* *communis*] | XP_002528196 | 1E-131 | 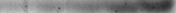 |
| M13-A6-1 | 318 | RNA binding motif protein [*Ricinus* *communis*] | XP_002520438 | 3E-35 | 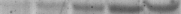 |
| M13-A6-2 | 175 | RNA binding protein, putative [*Ricinus* *communis*] | XP_002532732 | 1E-80 | 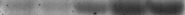 |
| M2-A8-5 | 145 | RNA-binding region-containing protein  [*Ricinus* *communis*] | XP_002523906 | 1E-15 | 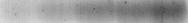 |
| M15-A10-4 | 322 | Auxin response factor, putative [*Ricinus* *communis*] | XP_002529941 | 1E-53 | 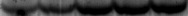 |
| M6-A6-2 | 255 | BTB/POZ domain-containing protein KCTD9, putative [*Ricinus* *communis*] | XP_002525705 | 5E-24 | 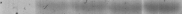 |
| M12-A7-3 | 204 | ethylene-responsive element-binding protein (EREBP) [*Citrus* *sinensis*] | ABM67696 | 3E-26 | 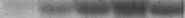 |
| M16-A6-1 | 406 | heat stress transcription factor [*Solanum* *peruvianum*] | CAA39034 | 2E-15 | 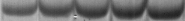 |
| M5-A9-2 | 339 | transcription regulator, putative [*Ricinus* *communis*] | XP_002515707 | 4E-44 | 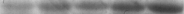 |
| M15-A8-4 | 167 | transcription factor btf3, putative [*Ricinus* *communis*] | XP_002523244 | 7E-61 | 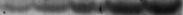 |
| M9-A9-4 | 232 | TIFY8 [*Arabidopsis* *thaliana*] | NP_567898 | 3E-15 | 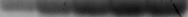 |
| M15-A12-4 | 96 | ccr4-associated factor, putative [*Ricinus* *communis*] | XP_002527306 | 1E-10 | 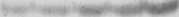 |
| M6-A12-6 | 147 | bel1 homeotic protein, putative [*Ricinus* *communis*] | XP_002529426 | 1E-149 | 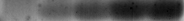 |
| M1-A8-3 | 198 | 40S ribosomal protein S17, putative  [*Ricinus* *communis*] | XP_002526912 | 4E-57 | 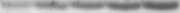 |
| M4-A12-6 | 79 | pseudouridine synthase and archaeosine  Transglycosylase-like protein [*Cucumis* *sativus*] | ACA24498 | 9E-33 | 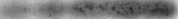 |
| M13-A7-1 | 341 | 60S ribosomal protein L24 [*Elaeis* *guineensis*] | ACF06439 | 1E-21 | 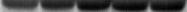 |
| M13-A10-5 | 85 | 60S ribosomal protein L34, putative  [*Ricinus* *communis*] | XP_002522429 | 2E-08 | 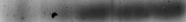 |
| M14-A8-3 | 192 | 60S ribosomal protein L32, putative  [*Ricinus* *communis*] | XP_002533495 | 3E-22 | 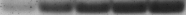 |
| M15-A7-1 | 537 | 60S acidic ribosomal protein P1 [*Ricinus* *communis*] | XP_002515071 | 3E-26 | 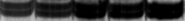 |
| M16-A11-4 | 211 | 60S ribosomal protein L13, putative  [*Ricinus* *communis*] | XP_002519996 | 4E-07 | 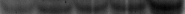 |
| M7-A12-1 | 129 | ribosomal protein L19 family protein  [*Arabidopsis* *thaliana*] | NP_567531 | 1E-39 | 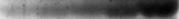 |
| M4-A11-2 | 431 | translation elongation factor 1-gamma  [*Prunus* *avium*] | AAG17901 | 0 | 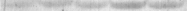 |
| **Protein degradation and storage (18)** | | | | | |
| M1-A7-10 | 105 | Diphthamide biosynthesis protein [*Ricinus* *communis*] | XP_002519128 | 2E-08 | 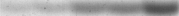 |
| M7-A9-1 | 552 | 26S proteasome alpha subunit [*Spinacia* *oleracea*] | BAA21651 | 3E-89 | 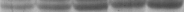 |
| M11-A5-1 | 269 | RNF5, putative [*Ricinus* *communis*] | XP_002510337 | 3E-15 | 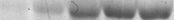 |
| M5-A5-4 | 287 | protein binding / ubiquitin-protein ligase  [*Vitis* *vinifera*] | XP_002280492 | 4E-30 | 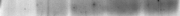 |
| M16-A11-5 | 125 | ubiquitin, putative [*Ricinus* *communis*] | XP_002513378 | 8E-05 | 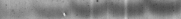 |
| M12-A11-1 | 431 | Ubiquitin[*Guzmania wittmackii* x *Guzmania lingulata*] | ACX46987 | 1E-41 | 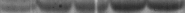 |
| M10-A7-3 | 125 | ubiquitin-protein ligase, putative [*Ricinus* *communis*] | XP_002530291 | 1E-134 | 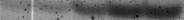 |
| M12-A5-5 | 92 | ubiquitin-conjugating enzyme variant [*Citrus* *sinensis*] | ACV49920 | 1E-10 | 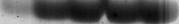 |
| M13-A6-3 | 155 | ubiquitin-like protein 5 [*Jatropha* *curcas*] | ACV70144 | 4E-34 | 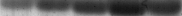 |
| M14-A8-4 | 143 | ubiquitin-conjugating enzyme m [*Ricinus* *communis*] | XP_002525098 | 6E-21 | 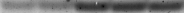 |
| M13-A9-2 | 239 | ubiquitin-conjugating enzyme E2 [*Ricinus* *communis*] | XP_002533452 | 3E-21 | 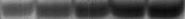 |
| M15-A6-5 | 179 | potential autophagy related protein-like [*Oryza* *sativa*] | BAD53524 | 2E-06 | 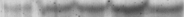 |
| M13-A11-3 | 153 | Cyclophilin [*Hevea* *brasiliensis*] | ABZ88806 | 8E-05 | 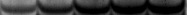 |
| M13-A12-2 | 253 | peptidylprolyl isomerase (cyclophilin)[*Betula* *pendula*] | CAC84116 | 4E-24 | 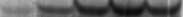 |
| M15-A6-8 | 85 | peptidase M1 family protein [*Arabidopsis* *thaliana*] | NP_001154442 | 4E-10 | 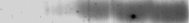 |
| M12-A10-3 | 237 | acetyltransferase complex ard1 subunit  [*Ricinus* *communis*] | XP_002517754 | 7E-90 | 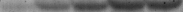 |
| M6-A8-2 | 395 | bifunctional dihydrofolate reductase-thymidylate synthase, putative [*Ricinus* *communis*] | XP_002512938 | 3E-53 | 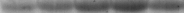 |
| M10-A10-2 | 345 | kelch repeat-containing F-box family protein  [*Arabidopsis* *thaliana*] | NP_198048 | 2E-31 | 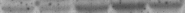 |
| **Transporters and intracellular transport (20)** | | | | | |
| M1-A6-5 | 202 | transporter, putative [*Ricinus* *communis*] | XP_002528763 | 1E-17 | 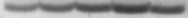 |
| M2-A7-5 | 203 | vacuolar ATP synthase subunit G [*Ricinus* *communis*] | XP_002529578 | 2E-18 | 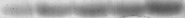 |
| M11-A7-1 | 428 | vacuolar ATP synthase proteolipid subunit 1, 2, 3, putative [*Ricinus* *communis*] | XP_002513751 | 9E-23 | 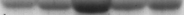 |
| M1-A8-5 | 87 | CAM7; calcium ion binding [*Arabidopsis* *thaliana*] | NP_189967 | 4E-79 | 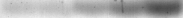 |
| M3-A11-4 | 377 | copper transport protein atox1 [*Ricinus* *communis*] | XP_002533872 | 4E-28 | 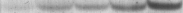 |
| M4-A9-2 | 340 | amino acid transporter family protein  [*Arabidopsis* *thaliana*] | NP_030664 | 4E-25 | 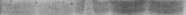 |
| M4-A12-4 | 192 | TRANSPORT INHIBITOR RESPONSE 1 protein,  putative [*Ricinus* *communis*] | XM_002512820 | 4E-50 | 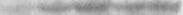 |
| M5-A11-1 | 227 | zinc/iron transporter, putative [*Ricinus* *communis*] | XP_002516442 | 3E-24 |  |
| M10-A8-1 | 360 | sugar transporter, putative [*Ricinus* *communis*] | XP_002528235 | 6E-22 |  |
| M10-A9-1 | 435 | sucrose transporter 3 [*Hevea* *brasiliensis*] | ABK60190 | 8E-61 |  |
| M16-A11-2 | 323 | ACD11; sphingosine transmembrane transporter  [*Arabidopsis* *thaliana*] | NP_181016 | 6E-22 |  |
| M3-A10-2 | 376 | nuclear transport factor, putative [*Ricinus* *communis*] | XP_002514900 | 2E-31 |  |
| M4-A10-4 | 117 | AP-4 complex subunit mu-1 [*Ricinus* *communis*] | XP_002510433 | 2E-09 |  |
| M9-A5-1 | 180 | sterol carrier, putative [*Ricinus* *communis*] | XP_002510217 | 5E-54 |  |
| M11-A8-3 | 323 | porin/voltage-dependent anion-selective channel protein [*Populus* *trichocarpa*] | XP_002324035 | 4E-36 |  |
| M15-A6-6 | 164 | ras-related protein Rab-6A [*Zea* *mays*] | NP_001150271 | 5E-99 |  |
| M9-A9-1 | 519 | AtRABA1f; GTP binding [*Arabidopsis* *thaliana*] | NP_200894 | 6E-66 |  |
| M5-A12-2 | 396 | copper-transporting atpase paa1 [*Ricinus* *communis*] | XP_002531490 | 4E-40 |  |
| M14-A10-5 | 281 | nonclathrin coat protein zeta1-COP [*Glycine* *max*] | BAA92779 | 1E-20 |  |
| M12-A7-1 | 333 | salt stress inducible small GTP binding protein Ran1 homolog [*Arabidopsis* *thaliana*] | AAB97312 | 3E-23 |  |
| **Cellular structure (7)** | | | | | |
| M1-A9-5 | 173 | actin depolymerizing factor, putative  [*Ricinus* *communis*] | XP_002533101 | 7E-70 |  |
| M1-A11-2 | 280 | UDP-glucuronosyltransferase [*Ricinus* *communis*] | XP_002514682 | 5E-13 |  |
| M7-A7-2 | 380 | NAD dependent epimerase/dehydratase  [*Ricinus* *communis*] | XP_002509687 | 5E-33 |  |
| M2-A7-1 | 493 | fiber protein Fb15 [*Gossypium* *barbadense*] | AAP34362| | 1E-37 |  |
| M6-A12-4 | 189 | hydroxyproline-rich glycoprotein family protein  [*Arabidopsis* *thaliana*] | NP_565899 | 2E-55 |  |
| M9-A8-3 | 307 | Pollen allergen Hev b 8.0101 [*Hevea* *brasiliensis*] | O65812 | 9E-25 |  |
| M12-A10-1 | 297 | Plastid-lipid-associated protein, chloroplast precursor,  putative [*Ricinus* *communis*] | XP_002524410 | 6E-14 |  |
| **Signal transduction (12)** | | | | | |
| M9-A8-2 | 402 | Protein-tyrosine phosphatase mitochondrial 1,  mitochondrial precursor, putative [*Ricinus* *communis*] | XP_002531467 | 5E-24 |  |
| M1-A6-2 | 432 | Glycogen synthase kinase-3 beta [*Ricinus* *communis*] | XP_002522231 | 8E-10 |  |
| M1-A7-8 | 163 | protein kinase atmrk1, putative [*Ricinus* *communis*] | XP_002524739 | 9E-25 |  |
| M2-A5-1 | 390 | casein protein kinase 2 alpha subunit [*Lolium* *perenne*] | BAD98470 | 2E-33 |  |
| M16-A9-4 | 171 | casein kinase II, alpha chain [*Ricinus* *communis*] | XP_002533161 | 2E-16 |  |
| M2-A5-2 | 354 | Protein kinase APK1B, chloroplast precursor, putative  [*Ricinus* *communis*] | XP_002527952 | 3E-20 |  |
| M2-A7-7 | 124 | calcium-binding protein, putative  [*Arabidopsis* *thaliana*] | NP_173259 | 1E-50 |  |
| M14-A10-9 | 223 | CBL-interacting protein kinase 2  [*Arabidopsis* *thaliana*] | AAF86506 | 3E-28 |  |
| M8-A6-2 | 223 | Phospholipase C 3 precursor [*Ricinus* *communis*] | XP_002524007 | 0 |  |
| M13-A8-7 | 151 | SWP (STRUWWELPETER) [*Arabidopsis* *thaliana*] | NP_187125 | 6E-09 |  |
| M5-A12-4 | 301 | early flowering 3 [*Mesembryanthemum* *crystallinum*] | AAQ73529 | 8E-12 |  |
| M13-A8-6 | 195 | phosphatidylinositol-4-phosphate 5-kinase, putative  [*Arabidopsis* *thaliana*] | AAM65200 | 8E-60 |  |
| **Stress and defense (23)** | | | | | |
| M6-A6-6 | 136 | trehalose-6-phosphate synthase [*Ricinus* *communis*] | XP_002531237 | 1E-06 |  |
| M1-A5-4 | 77 | Thioredoxin H-type, putative [*Ricinus* *communis*] | XP_002510456 | 2E-50 |  |
| M12-A10-4 | 209 | beta-1,3-glucanase [*Hevea* *brasiliensis*] | CAB38443 | 3E-20 |  |
| M1-A7-5 | 285 | Protein grpE, putative [*Ricinus* *communis*] | XP_002530954 | 6E-13 |  |
| M3-A11-3 | 411 | Glucan endo-1,3-beta-glucosidase precursor, putative  [*Ricinus* *communis*] | XP_002518468 | 6E-64 |  |
| M5-A8-1 | 485 | hydrogen peroxide-induced 1 [*Nicotiana* *tabacum*] | ACK38177 | 3E-19 |  |
| M6-A7-1 | 554 | latex-abundant protein [*Hevea* *brasiliensis*] | AAD13216 | 5E-62 |  |
| M7-A9-4 | 212 | Arylacetamide deacetylase, putative  [*Ricinus* *communis*] | XP_002523490 | 4E-20 |  |
| M6-A12-2 | 545 | disease resistance gene, putative[*Medicago* *truncatula*] | ABD33170 | 5E-31 |  |
| M10-A12-3 | 227 | programmed cell death, putative [*Ricinus* *communis*] | XP_002519736 | 2E-09 |  |
| M14-A9-3 | 228 | glutathione s-transferase, putative [*Ricinus* *communis*] | XP_002532823 | 5E-16 |  |
| M12-A5-4 | 157 | glutathione peroxidase, putative [*Ricinus* *communis*] | XP_002509791 | 8E-52 |  |
| M2-A10-3 | 292 | putative stress-induced protein[*Solanum* *commersonii*] | CAJ19269 | 1E-37 |  |
| M15-A12-2 | 150 | putative senescence-associated protein[*Pisum* *sativum*] | BAB33412 | 5E-08 |  |
| M12-A11-2 | 239 | glycine-rich RNA-binding protein [*Ricinus* *communis*] | XP_002526922 | 1E-37 |  |
| M13-A7-6 | 92 | NOI, putative [*Ricinus* *communis*] | XP_002532206 | 1E-14 |  |
| M13-A10-2 | 333 | Late embryogenesis abundant protein Lea14-A, putative [*Ricinus* *communis*] | XP_002511927 | 9E-12 |  |
| M13-A12-5 | 128 | heat-shock protein, putative [*Ricinus* *communis*] | XP_002530396 | 4E-73 |  |
| M14-A10-10 | 211 | Hydrophobic protein LTI6A [*Ricinus* *communis*] | XP_002512586 | 3E-11 |  |
| M9-A7-1 | 359 | lactoylglutathione lyase, putative [*Ricinus* *communis*] | XP_002517127 | 6E-16 |  |
| M16-A8-4 | 89 | beta-cyanoalanine synthase [*Hevea* *brasiliensis*] | AAP41852 | 1E-176 |  |
| M14-A9-1 | 456 | Regulatory protein NPR1, putative[*Ricinus* *communis*] | XP_002520549 | 3E-65 |  |
| M13-A5-3 | 165 | haloacid dehalogenase-like hydrolase family protein  [*Arabidopsis* *thaliana*] | NP_195843 | 4E-75 |  |
| **Secondary metabolism (8)** | | | | | |
| M16-A7-8 | 72 | s-adenosylmethionine decarboxylase  [*Ricinus* *communis*] | XP_002514534 | 3E-130 |  |
| M13-A8-4 | 301 | s-adenosylmethionine decarboxylase  [*Ricinus* *communis*] | XP_002525956 | 4E-30 |  |
| M2-A6-4 | 153 | cytochrome P450 monooxygenase CYP51G1  [*Medicago* *truncatula*] | ABC59074 | 2E-06 |  |
| M1-A10-3 | 174 | spermidine synthase 1, putative [*Ricinus* *communis*] | XP_002513571 | 3E-06 |  |
| M9-A6-1 | 403 | amine oxidase, putative [*Ricinus* *communis*] | XP_002521588 | 2E-47 |  |
| M11-A8-2 | 518 | biotin synthase, putative [*Ricinus* *communis*] | XP_002529753 | 4E-78 |  |
| M12-A9-5 | 116 | Amine oxidase [copper-containing] precursor, putative  [*Ricinus* *communis*] | XP_002516781 | 9E-125 |  |
| M15-A10-1 | 570 | Isochorismatase, putative [*Ricinus* *communis*] | XP_002530911 | 4E-56 |  |
| **Rubber biosynthesis (9)** | | | | | |
| M1-A6-7 | 138 | cis-prenyl transferase [*Hevea* *brasiliensis*] | AAX31281 | 2E-156 |  |
| M1-A10-2 | 197 | small rubber particle protein [*Hevea* *brasiliensis*] | AAC82355 | 4E-15 |  |
| M12-A6-4 | 134 | Small rubber particle protein [*Hevea* *brasiliensis*] | O82803.1 | 9E-07 |  |
| M14-A5-4 | 214 | rubber elongation factor [*Hevea* *brasiliensis*] | AAR11448 | 1E-18 |  |
| M13-A5-1 | 228 | rubber elongation factor [*Hevea* *brasiliensis*] | AAR11448 | 4E-11 |  |
| M2-A10-1 | 371 | rubber elongation factor [*Hevea* *brasiliensis*] | CAA39880 | 2E-30 |  |
| M11-A5-4 | 150 | farnesyl diphosphate synthase [*Hevea* *brasiliensis*] | AAM98379 | 0 |  |
| M16-A5-4 | 120 | hydroxymethylglutaryl coenzyme A synthase  [*Hevea* *brasiliensis*] | AAL18930 | 0 |  |
| M12-A9-3 | 209 | 3-hydroxy-3-methylglutaryl-coenzyme A reductase 2  [*Hevea* *brasiliensis*] | P29058 | 1E-20 |  |
| **Unclassified protein (7)** | | | | | |
| M5-A10-5 | 191 | Auxin-repressed 12.5 kDa protein [*Ricinus* *communis*] | XP_002509446 | 8E-45 |  |
| M16-A10-2 | 215 | ATGP4 [*Arabidopsis* *thaliana*] | NP_194229 | 9E-40 |  |
| M2-A7-3 | 309 | catalytic, putative [*Ricinus* *communis*] | XP_002533744 | 1E-30 |  |
| M16-A10-3 | 157 | nucleus protein [*Zea* *mays*] | ACG46992 | 4E-05 |  |
| M10-A12-5 | 147 | DNA binding [*Arabidopsis* *thaliana*] | NP_001030657 | 5E-11 |  |
| M3-A10-3 | 358 | TET2 (TETRASPANIN2) [*Arabidopsis* *thaliana*] | NP_179548 | 3E-23 |  |
| M14-A10-4 | 369 | ATP binding protein, putative [*Ricinus* *communis*] | XP_002517204 | 2E-23 |  |
| **Predicted protein (24)** | | | | | |
| M1-A6-3 | 303 | conserved hypothetical protein [*Ricinus communis*] | XP_002512047 | 3E-05 |  |
| M1-A8-2 | 200 | conserved hypothetical protein [*Ricinus communis*] | XP_002530241 | 2E-15 |  |
| M3-A6-2 | 308 | PREDICTED: hypothetical protein [*Vitis vinifera*] | XP_002280085 | 1E-23 |  |
| M3-A10-10 | 216 | hypothetical protein SORBIDRAFT_09g030890  [*Sorghum bicolor*] | XP_002441646 | 5E-12 |  |
| M4-A8-3 | 235 | conserved hypothetical protein [*Ricinus communis*] | XP_002524108 | 2E-17 |  |
| M5-A9-1 | 422 | conserved hypothetical protein [*Ricinus communis*] | XP_002509970 | 3E-23 |  |
| M6-A10-5 | 157 | predicted protein [*Populus trichocarpa*] | XP_002309483 | 2E-33 |  |
| M7-A5-6 | 75 | predicted protein [*Populus trichocarpa*] | XP_002326716 | 4E-51 |  |
| M7-A10-4 | 255 | PREDICTED: hypothetical protein [*Vitis vinifera*] | XP_002265352 | 4E-14 |  |
| M8-A9-1 | 419 | conserved hypothetical protein [*Ricinus communis*] | XP_002529468 | 8E-10 |  |
| M9-A8-5 | 190 | conserved hypothetical protein [*Ricinus communis*] | XP_002519688 | 9E-59 |  |
| M10-A9-5 | 169 | conserved hypothetical protein [*Ricinus communis*] | XP_002521967 | 6E-39 |  |
| M11-A5-2 | 296 | Unknown [*Glycine max*] | ACU15992 | 2E-08 |  |
| M11-A6-1 | 250 | conserved hypothetical protein [*Ricinus communis*] | XP_002519470 | 3E-25 |  |
| M12-A5-3 | 212 | predicted protein [*Populus trichocarpa*] | XP_002326304 | 2E-09 |  |
| M12-A7-6 | 105 | predicted protein [*Populus trichocarpa*] | XP_002327380 | 1E-33 |  |
| M13-A10-3 | 251 | conserved hypothetical protein [*Ricinus communis*] | XP_002515394 | 9E-30 |  |
| M13-A10-4 | 97 | conserved hypothetical protein [*Ricinus communis*] | XP_002510407 | 8E-46 |  |
| M15-A10-5 | 125 | conserved hypothetical protein [*Ricinus communis*] | XP_002520802 | 3E-52 |  |
| M14-A10-7 | 239 | conserved hypothetical protein [*Ricinus communis*] | XP_002512933 | 8E-13 |  |
| M8-A7-4 | 222 | PREDICTED: hypothetical protein [*Vitis vinifera*] | XP_002275093 | 2E-25 |  |
| M8-A12-1 | 319 | conserved hypothetical protein [*Ricinus communis*] | XP_002521549 | 1E-158 |  |
| M12-A12-1 | 229 | predicted protein [*Populus trichocarpa*] | XP_002316563 | 5E-43 |  |
| M16-A9-5 | 135 | predicted protein [*Populus trichocarpa*] | XP_002326716 | 4E-51 |  |
| **No hit sequence (14)** | | | | | |
| M1-A5-3 | 216 |  |  |  |  |
| M2-A5-8 | 154 |  |  |  |  |
| M3-A7-5 | 239 |  |  |  |  |
| M5-A9-4 | 235 |  |  |  |  |
| M6-A8-4 | 159 |  |  |  |  |
| M8-A11-3 | 184 |  |  |  |  |
| M12-A7-7 | 86 |  |  |  |  |
| M14-A10-11 | 165 |  |  |  |  |
| M15-A6-7 | 152 |  |  |  |  |
| M16-A5-5 | 76 |  |  |  |  |
| M16-A6-4 | 122 |  |  |  |  |
| M8-A12-3 | 157 |  |  |  |  |
| M8-A5-4 | 222 |  |  |  |  |

a): DE-TDFs number, including primer combinations for selective amplification. M: restriction enzyme *Mse* I, A: restriction enzyme *Apo* I. For example: M1-A5-1, using *Mse* I-SP_1_ and *Apo* I-SP_5_ selective primers for screening, obtaining the first DE-TDF.

b): DE-TDFs function annotation results in NCBI (<http://blast.ncbi.nlm.nih.gov/Blast.cgi>), “[ ]” is the corresponding species.

c): The expression pattern of DE-TDFs analyzed by cDNA-AFLP, showing the expression level in the first five tapping from left to right.
