# Supplementary material for: Transcript and Protein Profiling Provides Insights Into the Molecular Mechanisms of Harvesting-Induced Latex Production in Rubber Tree
Source: Front Genet. 2022 Feb 10;13:756270. doi: 10.3389/fgene.2022.756270 (PMC8869608; doi:10.3389/fgene.2022.756270)
Supplement: Supplementary file 5 [file Table5.DOC]

**Transcript and protein profiling provides insights into the molecular mechanisms of harvesting-induced latex production in rubber trees**

Yujie Fan^1, +^, Jiyan Qi^1, +^, Xiaohu Xiao^2, +^, Heping Li^1^, Jixian Lan^1^, Yacheng Huang^1^, Jianghua Yang^2^, Yi Zhang^1^, Shengmin Zhang^1^, Jun Tao^1^, Chaorong Tang^1,*^

^1^ Natural Rubber Cooperative Innovation Center of Hainan Province & Ministry of Education of PRC, Hainan University, Haikou 570228, China

^2^ Rubber Research Institute, Chinese Academy of Tropical Agricultural Sciences, Haikou 571101, China

^+^ These authors have contributed equally to this work.

^*^ Correspondence: [chaorongtang@126.com](mailto:chaorongtang@126.com); [chaorongtang@hainanu.edu.cn](mailto:chaorongtang@hainanu.edu.cn).

**Supplementary Table 5. qRT-PCR primers for latex regeneration related DE-TDFs and internal control *HbYLS8* gene**

| **DE-TDFs** | **Forward primer sequence (5’-3’)** | **Reverse primer sequence (5’-3’)** | **Length ^a)^ (bp)** |
| --- | --- | --- | --- |
| M1-A6-7 | GAGAACATGGAGTCGTATTCTGGA | TGCCAAAGTAAGTAGTTGCTCAGAC | 128 |
| M1-A10-2 | AGAATGTGGTGAAGACCGTGG | CTACCGAGTAAGTTTGGGCAGA | 153 |
| M2-A6-2 | TGGGAAAGGAAGGGTAAACAA | TGGGAAGGTGAAGCAGGAGAT | 133 |
| M2-A7-5 | TTGCTGCATCTATCTTGAGGTGA | GGAAAGTTACGGAGAGTAGCGG | 99 |
| M2-A9-3 | TAGACGTAATGCAGCCAAAAGC | GAAGGTGCCTGGAAAGATAAGC | 150 |
| M2-A10-1 | GCGGCAACTTATGCTGTGACTA | AACAACCGTGCTGTCTACAAACTT | 183 |
| M3-A8-4 | GGTGCATCAAGATAGCCAAAGC | ATAGACCCACAATCAACGCCTC | 136 |
| M3-A12-1 | AAACAATAACTGCCACTGAGGAATC | CGACAACACCTATCAGGCGACTA | 164 |
| M4-A5-5 | CTCCTGTACTCTTGGCAAAACG | GGAAACTGTGGCTGATGCTCTA | 183 |
| M4-A6-5 | AGGTCAATCTGCACTTCTGGAG | AGGTGAAGGCAAAGCAGGTTAC | 184 |
| M4-A12-5 | AGGATGAACATTGGAAGCCGT | CAAAGCCTAGCCATACTGGAAGA | 127 |
| M5-A5-1 | TCCTTCCGTTCCAAAATCCTC | GCAAATCGCTTCTTTCTACCC | 87 |
| M6-A6-6 | CAACCCACACTGCTCAAGAATG | GCATCGTCCTCTGGAAGAATAG | 115 |
| M7-A6-4 | CACAGCAGATGGCGTAAAACC | CGGGCAATGAAACTGATGAAC | 165 |
| M7-A9-5 | CACCTCGCCCACTTGTGATTA | TCTTCTTTGCCGTCTACTTTACTCT | 79 |
| M8-A5-6 | CAGGGTGCTTTCTTAGGGCT | CATTGTAGTGGCGATACTCAGGAT | 117 |
| M8-A8-3 | TCGGGAACGATTAGGAGACAA | CCAAGATCCTTTCACCTCAACC | 122 |
| M9-A9-1 | CAAATCTGAGCCTTGACGACCT | CCTTGAATCCAAATCCACCATC | 84 |
| M10-A8-1 | GGTACCTTTTTCCTATTTGCTGC | TCCTCTAAGATTTTCTCAACCTCCT | 113 |
| M11-A5-4 | CAAAGTTCTAAAGCCTTCACGAC | CGGGTGAGAATCTGGACAATC | 181 |
| M11-A7-1 | TGAAAGCCAGATAAGGAAACCG | GCCGCTGAAACCTGAAGACATA | 123 |
| M11-A11-5 | TCTTTCATCTGGTCTTGCTTGTG | GCTTTGGTTGTTGGGCATTAG | 104 |
| M12-A6-4 | ATTATGGCTGAAGAGGTGGAGG | CTTGGCATAAAGGTAGAGAGTTGAG | 108 |
| M12-A7-2 | CATCATCAATATGCGTTGGTAGGT | GGAAATGAAGATTCTCAGGCAACT | 169 |
| M12-A9-3 | GATCCAGCACAGAATGTTGAAAG | AGCAGACTGAGATGCAAGTTGAG | 147 |
| M13-A5-1 | AGGTTCCATGATGTTCCCAATAA | GAGCATAGCGAAGTACAAGAGTGAC | 85 |
| M13-A7-7 | GTTGGATTGGTGCTTTCTTATGG | ATTCTTGTAGCTTTGCGGCATT | 102 |
| M13-A8-2 | TGATTCTGGCAGCACTTACCG | CCTTTCTCATCCTTTCAACTGGG | 83 |
| M13-A11-4 | TCTGGCAGTGCTGTAAAGATAGGT | CTCCTGTTCGGATAATAGTTGCTG | 125 |
| M13-A11-5 | CCATAATCAAGTGTGCTTTCCG | CTGAAGCCACTGATGTTGCC | 148 |
| M14-A5-4 | GGCCATTTATTCTCATTATGAACA | GGAATGTAGGTGCCACACTCTG | 182 |
| M14-A7-4 | AGGTGCTGGTGACTCATTTGTAG | GCTCCTTTCTTGGTGGTTGTAA | 141 |
| M15-A6-6 | AGATTCAGAAGTCTCATCCCAAGC | GTCCACAAGGTCAGTTTTATTTCCA | 177 |
| M15-A7-3 | ACTCCCCTGCCTTGGTAAACT | TCCACTCAATGTGTTGCATCTTC | 148 |
| M15-A8-3 | GAATCTCACAATGACTGCTGCC | TTCTACCGGACTACATCCACAAAG | 160 |
| M16-A5-4 | GTCTTGCCTTCAACTTTCCTGC | GCGGGTGACACTGTTCTCTTATG | 137 |
| M16-A7-1 | TCCCAAGTTTCCCATCGTAATA | TTTTATGGCTGCTAACTGCTGC | 129 |
| *HbYLS8* | CCTCGTCGTCATCCGATTC | CAGGCACCTCAGTGATGTC | 131 |

a): The length of fragment amplified by qRT-PCR.
