# Supplementary material for: Transcript and Protein Profiling Provides Insights Into the Molecular Mechanisms of Harvesting-Induced Latex Production in Rubber Tree
Source: Front Genet. 2022 Feb 10;13:756270. doi: 10.3389/fgene.2022.756270 (PMC8869608; doi:10.3389/fgene.2022.756270)
Supplement: Supplementary file 6 [file DataSheet2.doc]

**Transcript and protein profiling provides insights into the molecular mechanisms of harvesting-induced latex production in rubber tree**

Yujie Fan^1, +^, Jiyan Qi^1, +^, Xiaohu Xiao^2, +^, Heping Li^1^, Jixian Lan^1^, Yacheng Huang^1^, Jianghua Yang^2^, Yi Zhang^1^, Shengmin Zhang^1^, Jun Tao^1^, Chaorong Tang^1,*^

^1^ Natural Rubber Cooperative Innovation Center of Hainan Province & Ministry of Education of PRC, Hainan University, Haikou 570228, China

^2^ Rubber Research Institute, Chinese Academy of Tropical Agricultural Sciences, Haikou 571101, China

^+^ These authors have contributed equally to this work.

^*^ Correspondence: [chaorongtang@126.com](mailto:chaorongtang@126.com); [chaorongtang@hainanu.edu.cn](mailto:chaorongtang@hainanu.edu.cn).


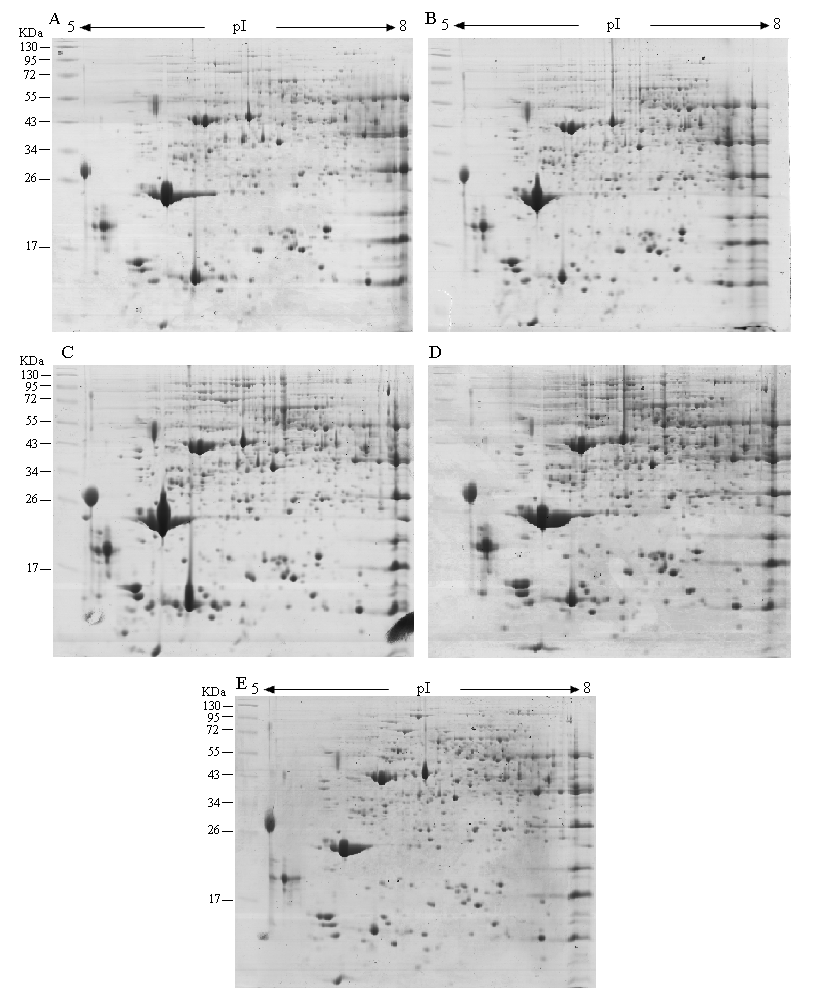


**Supplementary Figure 2. 2-DE images of latex C-serum proteins from reopened *Hevea* trees after a four-month resting period.** A: The latex protein of the first tapping; B: The second; C: The third; D: the forth; E: the fifth. kDa: Molecular weight of protein. pI: Protein isoelectric point.
