# Supplementary material for: Transcript and Protein Profiling Provides Insights Into the Molecular Mechanisms of Harvesting-Induced Latex Production in Rubber Tree
Source: Front Genet. 2022 Feb 10;13:756270. doi: 10.3389/fgene.2022.756270 (PMC8869608; doi:10.3389/fgene.2022.756270)
Supplement: Supplementary file 8 [file Table2.DOC]

**Transcript and protein profiling provides insights into the molecular mechanisms of harvesting-induced latex production in rubber trees**

Yujie Fan^1, +^, Jiyan Qi^1, +^, Xiaohu Xiao^2, +^, Heping Li^1^, Jixian Lan^1^, Yacheng Huang^1^, Jianghua Yang^2^, Yi Zhang^1^, Shengmin Zhang^1^, Jun Tao^1^, Chaorong Tang^1,*^

^1^ Natural Rubber Cooperative Innovation Center of Hainan Province & Ministry of Education of PRC, Hainan University, Haikou 570228, China

^2^ Rubber Research Institute, Chinese Academy of Tropical Agricultural Sciences, Haikou 571101, China

^+^ These authors have contributed equally to this work.

^*^ Correspondence: [chaorongtang@126.com](mailto:chaorongtang@126.com); [chaorongtang@hainanu.edu.cn](mailto:chaorongtang@hainanu.edu.cn).

**Supplementary Table 2. Functional annotation and cDNA-AFLP profiles of the down-regulated DE-TDFs**

| **DE-TDFs ^a)^** | **Size**  **(bp)** | **Function annotation ^b)^[species]** | **Accession number** | **E-Value** | **cDNA-AFLP gel picture ^c)^** |
| --- | --- | --- | --- | --- | --- |
| **Primary metabolism (12)** | | | | | |
| M6-A10-1 | 648 | Beta-amylase, putative [*Ricinus* *communis*] | XP_002518196 | 3E-101 | 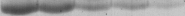 |
| M1-A11-3 | 263 | glycerate dehydrogenase, putative  [*Ricinus* *communis*] | XP_002511757 | 2E-17 | 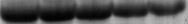 |
| M6-A6-5 | 395 | bifunctional dihydrofolate reductase-thymidylate synthase[*Ricinus* *communis*] | XP_002512938 | 3E-53 | 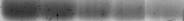 |
| M8-A10-5 | 191 | 3-5 exonuclease, putative [*Ricinus* *communis*] | XP_002527682 | 6E-31 | 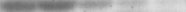 |
| M13-A7-5 | 130 | 3'-5' exonuclease, putative [*Ricinus* *communis*] | XP_002525702 | 6E-79 | 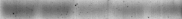 |
| M4-A11-4 | 259 | threonine dehydratase/deaminase [*Ricinus* *communis*] | XP_002528625 | 5E-29 | 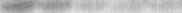 |
| M16-A12-3 | 292 | 1-acyl-sn-glycerol-3-phosphate acyltransferase zeta  precursor, putative [*Ricinus* *communis*] | XP_002525812 | 2E-39 | 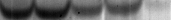 |
| M9-A5-4 | 76 | Dolichyl-phosphate beta-glucosyltransferase, putative  [*Ricinus* *communis*] | XP_002530857 | 1E-141 | 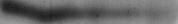 |
| M13-A8-5 | 264 | adenosylhomocysteinase, putative [*Ricinus* *communis*] | XP_002522805 | 0 | 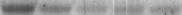 |
| M6-A8-6 | 102 | cysteine desulfurylase, putative [*Ricinus* *communis*] | XP_002531989 | 0 | 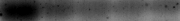 |
| M16-A10-4 | 114 | short-chain dehydrogenase/reductase family protein  [*Arabidopsis* *thaliana*] | NP_186983 | 6E-31 | 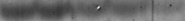 |
| M2-A6-3 | 339 | 3,4-dihydroxy-2-butanonekinase[*Solanumlycopersicum*] | CAA72805 | 6E-08 | 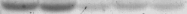 |
| **Energy (5)** | | | | | |
| M2-A9-3 | 337 | adenylate kinase 1 chloroplast [*Ricinus* *communis*] | XP_002511210 | 2E-16 | 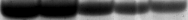 |
| M13-A11-4 | 416 | mitochondrial pyruvate dehydrogenase kinase isoform 2  [*Pisum* *sativum*] | ABW03160 | 3E-24 | 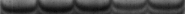 |
| M14-A10-6 | 258 | zinc binding dehydrogenase [*Ricinus* *communis*] | XP_002527341 | 1E-07 | 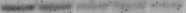 |
| M11-A9-4 | 150 | electron transporter, putative [*Ricinus* *communis*] | XP_002513069 | 8E-10 | 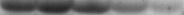 |
| M4-A12-5 | 115 | Phosphoenolpyruvate carboxylase [*Ricinus* *communis*] | XP_002530381 | 1E-136 | 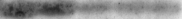 |
| **Cell growth and division (5)** | | | | | |
| M3-A7-4 | 264 | Proliferating-cell nucleolar antigen p120  [*Ricinus* *communis*] | XP_002522787 | 1E-160 | 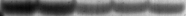 |
| M13-A12-3 | 158 | DNA double-strand break repair rad50 ATPase, putative  [*Ricinus* *communis*] | XP_002533906 | 1E-11 | 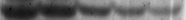 |
| M12-A10-2 | 254 | Protein COBRA precursor, putative [*Ricinus* *communis*] | XP_002514629 | 2E-11 | 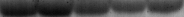 |
| M7-A5-5 | 104 | smg-7, putative [*Ricinus* *communis*] | XP_002526042 | 0 | 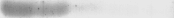 |
| M11-A10-5 | 93 | DNA repair protein xp-E, putative [*Ricinus* *communis*] | XP_002526608 | 1E-08 | 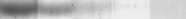 |
| **Transcription and protein synthesis (35)** | | | | | |
| M1-A5-1 | 629 | AP2 domain-containing transcription factor  [*Populus* *trichocarpa*] | XP_002308938 | 1E-26 | 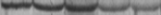 |
| M14-A7-5 | 131 | putative AP2-binding protein [*Jatropha* *curcas*] | AAZ14831 | 1E-21 | 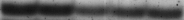 |
| M1-A7-1 | 552 | Ubiquitin-protein ligase BRE1A [*Ricinus* *communis*] | XP_002512311 | 5E-53 | 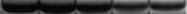 |
| M1-A9-3 | 307 | ccaat-binding transcription factor [*Ricinus* *communis*] | XP_002513360 | 7E-11 | 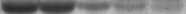 |
| M2-A10-4 | 229 | RNA splicing protein mrs2 [*Ricinus* *communis*] | XP_002517021 | 2E-06 | 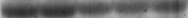 |
| M3-A7-1 | 506 | chromatin remodeling complex subunit  [*Populus* *trichocarpa*] | XP_002315568 | 1E-52 | 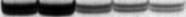 |
| M9-A7-3 | 224 | chromatin remodeling complex subunit  [*Populus* *trichocarpa*] | XP_002306648 | 4E-28 | 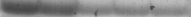 |
| M9-A8-1 | 619 | chromatin remodeling complex subunit  [*Populus* *trichocarpa*] | XP_002300622 | 6E-45 | 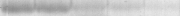 |
| M5-A12-6 | 169 | Transcription elongation factor B polypeptide 1  [*Anoplopoma* *fimbria*] | ACQ57881 | 2E-21 | 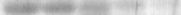 |
| M8-A6-3 | 202 | Pre-rRNA-processing protein TSR2[*Ricinus* *communis*] | XP_002512262 | 2E-09 | 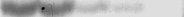 |
| M8-A12-2 | 187 | Protein ULTRAPETALA1, putative [*Ricinus* *communis*] | XP_002517976 | 1E-23 | 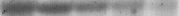 |
| M10-A9-4 | 203 | nuclear acid binding protein [*Ricinus* *communis*] | XP_002518798 | 5E-135 | 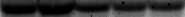 |
| M11-A6-3 | 135 | heterogeneous nuclear ribonucleoprotein  [*Ricinus* *communis*] | XP_002513501 | 1E-115 | 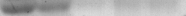 |
| M9-A6-3 | 184 | Stromal membrane-associated protein  [*Ricinus* *communis*] | XP_002531044 | 4E-07 | 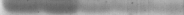 |
| M1-A6-4 | 241 | RNA binding protein, putative [*Ricinus* *communis*] | XP_002513779 | 5E-65 | 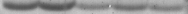 |
| M1-A7-6 | 173 | yth domain-containing protein [*Ricinus* *communis*] | XP_002513402 | 0 | 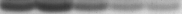 |
| M1-A12-1 | 209 | bromodomain-containing protein [*Ricinus* *communis*] | XP_002519772 | 5E-10 | 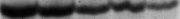 |
| M9-A8-7 | 148 | PREDICTED: Myc2 bHLH protein [*Vitis* *vinifera*] | XP_002264409 | 0 | 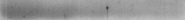 |
| M14-A9-2 | 438 | 116 kD U5 small nuclear ribonucleoprotein component,  putative [*Ricinus* *communis*] | XP_002532501 | 1E-74 | 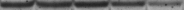 |
| M14-A12-1 | 639 | Poly(rC)-binding protein, putative [*Ricinus* *communis*] | XP_002519648 | 7E-64 | 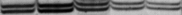 |
| M8-A10-4 | 229 | Pinin, putative [*Ricinus* *communis*] | XP_002522077 | 2E-28 | 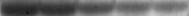 |
| M10-A11-1 | 580 | something about silencing protein sas10 [*Ricinus* *communis*] | XP_002527367 | 2E-39 | 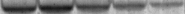 |
| M7-A11-2 | 167 | UBP1 interacting protein 2a [*Arabidopsis* *thaliana*] | NP_567042 | 5E-09 | 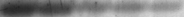 |
| M7-A11-1 | 292 | NAF1 [*Arabidopsis* *thaliana*] | NP_171852 | 9E-41 | 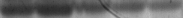 |
| M11-A6-5 | 111 | snRNA-activating protein complex subunit, putative  [*Ricinus* *communis*] | XP_002525235 | 1E-105 | 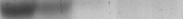 |
| M2-A12-5 | 143 | dead box ATP-dependent RNA helicase  [*Ricinus* *communis*] | XP_002520584 | 3E-14 | 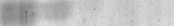 |
| M7-A10-3 | 362 | translation elongation factor [*Ricinus* *communis*] | XP_002515715 | 3E-46 | 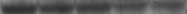 |
| M10-A6-1 | 321 | eukaryotic translation elongation factor  [*Ricinus* *communis*] | XP_002513404 | 2E-07 | 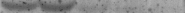 |
| M9-A11-3 | 298 | ribosome biogenesis regulatory protein  [*Ricinus* *communis*] | XP_002524474 | 7E-19 | 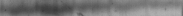 |
| M10-A12-7 | 80 | Eukaryotic translation initiation factor 3 subunit, putative [*Ricinus* *communis*] | XP_002512475 | 5E-20 | 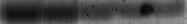 |
| M8-A9-2 | 336 | translation initiation factor if-2 [*Ricinus* *communis*] | XP_002528094 | 0 | 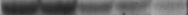 |
| M4-A11-5 | 105 | isoleucyl tRNA synthetase [*Ricinus* *communis*] | XP_002529754 | 1E-104 | 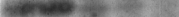 |
| M13-A11-6 | 294 | poly-A binding protein, putative [*Ricinus* *communis*] | XP_002513178 | 1E-16 | 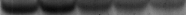 |
| M1-A6-8 | 90 | TIF3H1 [*Arabidopsis* *thaliana*] | NP_563880 | 1E-158 | 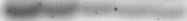 |
| M7-A10-2 | 446 | nucleic acid binding protein [*Ricinus* *communis*] | XP_002532850 | 2E-15 | 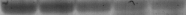 |
| **Protein degradation and storage (9)** | | | | | |
| M6-A8-3 | 311 | protein disulfide isomerase [*Malus* x *domestica*] | AAV50008 | 3E-27 | 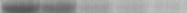 |
| M8-A11-1 | 436 | chaperonin containing t-complex protein 1, gamma subunit,tcpg, putative [*Ricinus* *communis*] | XP_002529662 | 4E-46 | 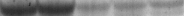 |
| M12-A8-2 | 334 | ubiquitin-protein ligase, putative [*Ricinus* *communis*] | XP_002513950 | 6E-29 | 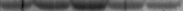 |
| M14-A7-3 | 178 | HEAT repeat-containing protein [*Arabidopsis* *thaliana*] | NP_181605 | 1E-15 | 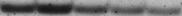 |
| M15-A11-2 | 159 | sorting and assembly machinery (sam50) protein,  putative [*Ricinus* *communis*] | XP_002520530 | 5E-08 | 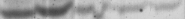 |
| M13-A8-3 | 444 | scythe/bat3, putative [*Ricinus* *communis*] | XP_002532506 | 2E-47 | 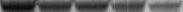 |
| M7-A5-4 | 130 | Minor histocompatibility antigen H13  [*Ricinus* *communis*] | XP_002529686 | 1E-141 | 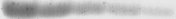 |
| M4-A7-3 | 144 | chaperonin 60 [*Dictyostelium* *discoideum*] | AAB17277 | 1E-179 | 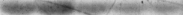 |
| M13-A11-7 | 175 | cysteine-type endopeptidase/ubiquitin thiolesterase  [*Zea* *mays*] | NP_001149375 | 9E-80 | 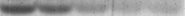 |
| **Transporters and intracellular transport (18)** | | | | | |
| M1-A11-1 | 369 | cation efflux protein/ zinc transporter  [*Ricinus* *communis*] | XP_002533664 | 3E-22 | 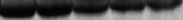 |
| M9-A10-1 | 414 | cation efflux protein/ zinc transporter  [*Ricinus* *communis*] | XP_002533664 | 9E-44 | 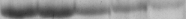 |
| M2-A9-2 | 321 | ATP-dependent transporter [*Ricinus* *communis*] | XP_002523695 | 8E-11 | 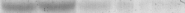 |
| M7-A6-4 | 343 | vacuolar ATP synthase subunit h [*Ricinus* *communis*] | XP_002520072 | 2E-32 | 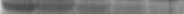 |
| M9-A11-1 | 441 | copper-transporting atpase p-type [*Ricinus* *communis*] | XP_002509783 | 1E-63 | 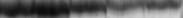 |
| M11-A6-2 | 213 | ATP-binding cassette transporter [*Ricinus* *communis*] | XP_002527329 | 3E-21 | 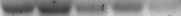 |
| M1-A10-6 | 120 | similar to ADP-ribosylation factor [*Vitis* *vinifera*] | XP_002277054 | 9E-54 | 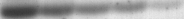 |
| M2-A10-2 | 367 | Patellin-3, putative [*Ricinus* *communis*] | XP_002521801 | 1E-32 | 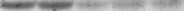 |
| M8-A5-2 | 402 | Protein SEY1, putative [*Ricinus* *communis*] | XP_002527405 | 8E-32 | 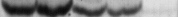 |
| M12-A11-4 | 92 | Vesicle-associated membrane protein  [*Ricinus* *communis*] | XP_002516908 | 1E-20 | 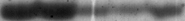 |
| M7-A10-8 | 95 | Vesicle-associated membrane protein  [*Ricinus* *communis*] | XP_002530145 | 9E-102 | 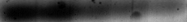 |
| M9-A9-5 | 200 | nodulin family protein [*Arabidopsis* *thaliana*] | NP_177616 | 2E-08 | 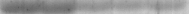 |
| M4-A6-5 | 140 | RAN3 [*Arabidopsis* *thaliana*] | NP_200330 | 3e-118 | 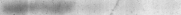 |
| M15-A9-1 | 391 | guanine nucleotide-exchange [*Ricinus* *communis*] | XP_002516179 | 1E-38 | 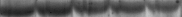 |
| M2-A7-6 | 128 | RAB1A [*Lotus* *japonicus*] | CAA66447 | 3E-13 | 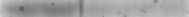 |
| M13-A7-7 | 76 | Prenylated Rab acceptor protein [*Ricinus* *communis*] | XP_002531279 | 2E-11 | 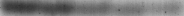 |
| M10-A6-4 | 148 | interferon-induced guanylate-binding protein, putative  [*Ricinus* *communis*] | XP_002509420 | 0 | 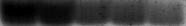 |
| M3-A7-8 | 160 | ATATH9, putative [*Ricinus* *communis*] | XP_002533645 | 3E-16 | 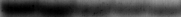 |
| **Cellular structure (6)** | | | | | |
| M6-A10-4 | 172 | alpha-tubulin [*Gossypium* *hirsutum*] | ABO47735 | 0 | 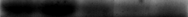 |
| M4-A5-8 | 233 | pectin acetylesterase, putative [*Ricinus* *communis*] | XP_002532332 | 2E-32 | 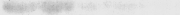 |
| M11-A10-2 | 150 | phosphoric diester hydrolase [*Ricinus* *communis*] | XP_002510406 | 1E-15 | 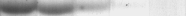 |
| M15-A12-3 | 146 | beta-1,3-glucuronyltransferase [*Ricinus* *communis*] | XP_002524911 | 6E-57 | 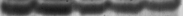 |
| M1-A7-4 | 348 | hydroxyproline-rich glycoprotein family protein  [*Arabidopsis* *thaliana*] | NP_569016 | 3E-23 | 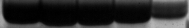 |
| M12-A9-4 | 121 | fiber protein Fb34 [*Zea* *mays*] | |NP_001148657 | 6E-51 | 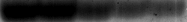 |
| **Signal transduction (18)** | | | | | |
| M4-A11-1 | 577 | WD-repeat protein, putative [*Ricinus* *communis*] | XP_002511220 | 3E-52 | 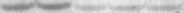 |
| M16-A5-1 | 504 | WD-repeat protein, putative [*Ricinus* *communis*] | XP_002527178 | 1E-28 | 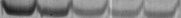 |
| M15-A6-4 | 188 | Phospholipase A22 [*Ricinus* *communis*] | XP_002526043 | 1E-105 | 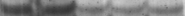 |
| M11-A8-8 | 81 | snf1-kinase beta subunit, plants [*Ricinus* *communis*] | XP_002515602 | 6E-06 | 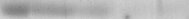 |
| M13-A5-2 | 164 | casein kinase, putative [*Ricinus* *communis*] | XP_002521378 | 2E-21 | 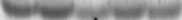 |
| M13-A11-1 | 623 | leucine-rich repeat transmembrane protein kinase,  putative [*Ricinus* *communis*] | XP_002533323 | 2E-85 | 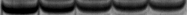 |
| M15-A5-3 | 79 | protein phosphatase 2c, putative [*Ricinus* *communis*] | XP_002521368 | 1E-90 | 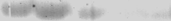 |
| M16-A12-5 | 110 | calcium-dependent protein kinase [*Ricinus* *communis*] | XP_002521658 | 1E-116 | 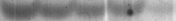 |
| M4-A12-1 | 441 | Auxin-binding protein T85 precursor  [*Ricinus* *communis*] | XP_002524798 | 5E-76 | 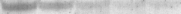 |
| M14-A10-8 | 228 | Protein SIS1, putative [*Ricinus* *communis*] | XP_002517853 | 3E-94 | 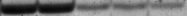 |
| M7-A7-1 | 563 | UBX domain-containing protein [*Ricinus* *communis*] | XP_002521873 | 4E-51 |  |
| M11-A9-8 | 106 | Cyclin-dependent kinases regulatory subunit, putative  [*Ricinus* *communis*] | XP_002523737 | 2E-35 |  |
| M1-A10-5 | 151 | steroid binding protein, putative [*Ricinus* *communis*] | XP_002510808 | 1E-85 |  |
| M9-A5-2 | 177 | Nucleoprotein TPR, putative [*Ricinus* *communis*] | XP_002518821 | 5E-99 |  |
| M6-A11-6 | 179 | Cyclic phosphodiesterase, putative [*Ricinus* *communis*] | XP_002522285 | 9E-59 |  |
| M5-A5-5 | 150 | MAP3Ka [*Lycopersicon* *esculentum*] | AAS78640 | 3E-19 |  |
| M6-A5-2 | 462 | bypass1 [*Nicotiana* *benthamiana*] | ABX26124 | 1E-22 |  |
| M8-A5-3 | 279 | phospholipid/glycerol acyltransferase family protein  [*Arabidopsis* *thaliana*] | NP_565249 | 1E-132 |  |
| **Stress and defense (16)** | | | | | |
| M3-A8-2 | 338 | putative Phage-like protein [*Acinetobacter* *baumannii* *AYE*] | YP_001712512 | 2E-15 |  |
| M3-A12-4 | 233 | heat shock protein 15 [*Acinetobacter* *sp*. RUH2624] | ZP_05826500 | 1E-35 |  |
| M10-A9-2 | 430 | heat shock protein [*Hevea* *brasiliensis*] | AAQ08597 | 3E-52 |  |
| M5-A10-3 | 281 | osmotin-like protein I [*Gossypium* *hirsutum*] | AAQ74156 | 6E-10 |  |
| M6-A5-4 | 195 | cysteine protease, putative [*Ricinus* *communis*] | XP_002510469 | 6E-73 |  |
| M9-A10-4 | 140 | Protein MLO, putative [*Ricinus* *communis*] | XP_002533335 | 1E-115 |  |
| M6-A10-7 | 129 | senescence-related protein [*Camellia* *sinensis*] | ACH87168 | 6E-104 |  |
| M8-A7-2 | 513 | latex-abundant protein [*Hevea* *brasiliensis*] | AAD13216 | 7E-61 |  |
| M15-A6-2 | 438 | disease resistance protein [*Populus* *trichocarpa*] | XP_002322142 | 3E-43 |  |
| M15-A11-1 | 201 | PR-4 type protein [*Vitis* *vinifera*] | AAC33732 | 7E-20 |  |
| M16-A9-2 | 438 | Zeamatin precursor, putative [*Ricinus* *communis*] | XP_002534154 | 2E-23 |  |
| M6-A9-1 | 321 | DnaJ protein [*Hevea* *brasiliensis*] | AAD12055 | 2E-46 |  |
| M9-A10-5 | 99 | Chaperone protein dnaJ, putative [*Ricinus* *communis*] | XP_002514419 | 3E-89 |  |
| M3-A5-2 | 150 | Disease resistance protein RPP8 [*Ricinus* *communis*] | XP_002524237 | 2E-55 |  |
| M1-A7-9 | 110 | 5-oxoprolinase, putative [*Ricinus* *communis*] | XP_002527743 | 2E-10 |  |
| M13-A12-4 | 164 | ripening-related protein-like [*Vitis* *vinifera*] | XP_002267559 | 1E-95 |  |
| **Secondary metabolism (2)** | | | | | |
| M12-A6-2 | 818 | Nitrilase, putative [*Ricinus* *communis*] | XP_002523857 | 2E-126 |  |
| M3-A8-6 | 191 | membrane bound O-acyl transferase (MBOAT) family protein[*Arabidopsis* *thaliana*] | NP_172724 | 1E-172 |  |
| **Unclassified proteins (16)** | | | | | |
| M4-A7-2 | 216 | ATP binding protein, putative [*Ricinus* *communis*] | XP_002526190 | 3E-44 |  |
| M15-A9-2 | 199 | ATP binding protein, putative [*Ricinus* *communis*] | XP_002510881 | 2E-11 |  |
| M16-A12-6 | 98 | ATP binding protein, putative [*Ricinus* *communis*] | XP_002529486 | 0 |  |
| M5-A8-2 | 477 | DUF593-containing protein 2 [*Medicago* *truncatula*] | ABY71162 | 5E-12 |  |
| M10-A9-6 | 98 | PMP, putative [*Ricinus* *communis*] | XP_002518115 | 1E-129 |  |
| M3-A6-4 | 277 | ankyrin repeat-containing protein [*Ricinus* *communis*] | XP_002519454 | 2E-83 |  |
| M8-A7-3 | 309 | ankyrin repeat protein-like [*Oryza* *sativa* *Japonica* *Group*] | BAD19146 | 1E-41 |  |
| M11-A10-1 | 584 | ankyrin repeat-containing protein [*Ricinus* *communis*] | XP_002519454 | 5E-83 |  |
| M16-A7-3 | 205 | zinc ion binding protein, putative [*Ricinus* *communis*] | XP_002511320 | 3E-105 |  |
| M3-A8-3 | 301 | Transmembrane protein, putative [*Ricinus* *communis*] | XP_002525329 | 6E-23 |  |
| M11-A10-4 | 113 | Membrane protein PB1A10.07c [*Ricinus* *communis*] | XP_002512768 | 5E-15 |  |
| M8-A5-5 | 189 | endonuclease reverse transcriptase [*Bos* *taurus*] | XP_001789053 | 4E-15 |  |
| M10-A7-2 | 196 | branched-chain amino acid transport system II carrier  protein[*Acinetobacter* *sp*. RUH2624] | ZP_05824568 | 3E-06 |  |
| M6-A5-1 | 580 | PAS/PAC sensor protein  [*Geobacter* *uraniireducens* Rf4] | YP_001231648 | 8E-07 |  |
| M8-A5-7 | 169 | TET10 (TETRASPANIN10) [*Arabidopsis* *thaliana*] | NP_974077 | 5E-78 |  |
| M1-A8-4 | 134 | putative Glu-rich protein [*Medicago* *truncatula*] | ABQ23583 | 8E-39 |  |
| **Predicted proteins (20)** | | | | | |
| M3-A9-2 | 299 | conserved hypothetical protein [*Ricinus communis*] | XP_002527655 | 2E-110 |  |
| M4-A10-2 | 230 | conserved hypothetical protein [*Ricinus communis*] | XP_002519827 | 5E-15 |  |
| M6-A11-5 | 283 | conserved hypothetical protein [*Ricinus communis*] | XP_002510479 | 2E-05 |  |
| M7-A9-6 | 145 | PREDICTED: hypothetical protein [*Vitis vinifera*] | XP_002279853 | 5E-06 |  |
| M8-A8-2 | 192 | hypothetical protein AbauAB_07292  [*Acinetobacter baumannii AB900*] | ZP_04661413 | 5E-09 |  |
| M10-A8-5 | 84 | conserved hypothetical protein [*Ricinus communis*] | XP_002528217 | 9E-98 |  |
| M10-A9-3 | 273 | conserved hypothetical protein [*Ricinus communis*] | XP_002520310 | 7E-28 |  |
| M11-A8-1 | 676 | predicted protein [*Populus trichocarpa*] | XP_002317331 | 3E-99 |  |
| M11-A10-3 | 117 | conserved hypothetical protein [*Ricinus communis*] | XP_002517027 | 5E-95 |  |
| M12-A5-1 | 878 | unnamed protein product [*Homo sapiens*] | BAG64005 | 7E-71 |  |
| M13-A7-2 | 283 | conserved hypothetical protein [*Ricinus communis*] | XP_002532150 | 2E-31 |  |
| M13-A7-3 | 213 | predicted protein [*Populus trichocarpa*] | XP_002326826 | 3E-11 |  |
| M13-A9-1 | 632 | conserved hypothetical protein [*Ricinus communis*] | XP_002532552 | 4E-43 |  |
| M6-A12-7 | 118 | predicted protein, mRNA [*Populus trichocarpa*] | XM_002300944 | 3E-19 |  |
| M9-A10-3 | 245 | PREDICTED: hypothetical protein [*Vitis vinifera*] | XP_002264749 | 4E-55 |  |
| M5-A11-4 | 135 | predicted protein [*Populus trichocarpa*] | XP_002322819 | 5E-11 |  |
| M15-A10-2 | 383 | predicted protein [*Populus trichocarpa*] | XP_002319603 | 3E-20 |  |
| M7-A10-7 | 170 | PREDICTED: hypothetical protein [*Vitis vinifera*] | XP_002265330 | 1E-156 |  |
| M7-A5-3 | 263 | conserved hypothetical protein [*Ricinus communis*] | XP_002514251 | 0 |  |
| M11-A10-6 | 76 | conserved hypothetical protein [*Ricinus communis*] | XP_002518581 | 4E-29 |  |
| **No hit sequence (18)** | | | | | |
| M1-A6-6 | 175 |  |  |  |  |
| M2-A8-4 | 166 |  |  |  |  |
| M2-A9-5 | 151 |  |  |  |  |
| M4-A7-4 | 133 |  |  |  |  |
| M5-A11-3 | 182 |  |  |  |  |
| M6-A7-3 | 196 |  |  |  |  |
| M6-A9-2 | 143 |  |  |  |  |
| M6-A9-3 | 91 |  |  |  |  |
| M8-A6-4 | 193 |  |  |  |  |
| M8-A9-4 | 191 |  |  |  |  |
| M9-A6-2 | 265 |  |  |  |  |
| M10-A5-2 | 165 |  |  |  |  |
| M12-A10-6 | 106 |  |  |  |  |
| M14-A12-2 | 226 |  |  |  |  |
| M5-A8-3 | 308 |  |  |  |  |
| M6-A10-6 | 140 |  |  |  |  |
| M8-A6-1 | 317 |  |  |  |  |
| M7-A8-3 | 172 |  |  |  |  |

a): DE-TDFs number, including primer combinations for selective amplification. M: restriction enzyme *Mse* I, A: restriction enzyme *Apo* I. For example: M1-A5-1, using *Mse* I-SP_1_ and *Apo* I-SP_5_ selective primers for screening, obtaining the first DE-TDF.

b): DE-TDFs function annotation results in NCBI (<http://blast.ncbi.nlm.nih.gov/Blast.cgi>), “[ ]” is the corresponding species.

c): The expression pattern of DE-TDFs analyzed by cDNA-AFLP, showing the expression level in the first five tapping from left to right.
